# Modelling the impact of ivermectin on River Blindness and its burden of morbidity and mortality in African Savannah: EpiOncho projections

**DOI:** 10.1186/1756-3305-7-241

**Published:** 2014-05-26

**Authors:** Hugo C Turner, Martin Walker, Thomas S Churcher, María-Gloria Basáñez

**Affiliations:** 1 Department of Infectious Disease Epidemiology, School of Public Health, Faculty of Medicine (St. Mary’s Campus), Imperial College London, Norfolk Place, London W2 1PG, UK

**Keywords:** Neglected tropical diseases, River blindness, Onchocerciasis, Africa, Epidemiology, Control programmes, Ivermectin, Mathematical modelling, Disease burden, DALY, Morbidity, Mortality

## Abstract

**Background:**

The African Programme for Onchocerciasis Control (APOC) has refocused its goals on the elimination of infection where possible, seemingly achievable by 15–17 years of annual mass distribution of ivermectin in some African foci. Previously, APOC had focused on the elimination of onchocerciasis as a public health problem. Timeframes have been set by the World Health Organization, the London Declaration on Neglected Tropical Diseases and the World Bank to achieve these goals by 2020–2025.

**Methods:**

A novel mathematical model of the dynamics of onchocercal disease is presented which links documented associations between *Onchocerca volvulus* infection and the prevalence and incidence of morbidity and mortality to model outputs from our host age- and sex-structured onchocerciasis transmission framework (EpiOncho). The model is calibrated for African savannah settings, and used to assess the impact of long-term annual mass administration of ivermectin on infection and ocular and skin disease and to explore how this depends on epidemiological and programmatic variables.

**Results:**

Current onchocerciasis disease projections, which do not account for excess mortality of sighted individuals with heavy microfilarial loads, underestimate disease burden. Long-term annual ivermectin treatment is highly effective at reducing both the morbidity and mortality associated with onchocerciasis, and this result is not greatly influenced by treatment coverage and compliance. By contrast, impact on microfilarial prevalence and intensity is highly dependent on baseline endemicity, treatment coverage and systematic non-compliance.

**Conclusions:**

The goals of eliminating morbidity and infection with ivermectin alone are distinctly influenced by epidemiological and programmatic factors. Whilst the former goal is most certainly achievable, reaching the latter will strongly depend on initial endemicity (the higher the endemicity, the greater the magnitude of inter-treatment transmission), advising caution when generalising the applicability of successful elimination outcomes to other areas. The proportion of systematic non-compliers will become far more influential in terms of overall success in achieving elimination goals.

## Background

Human onchocerciasis is also known as ‘river blindness’ because the simuliid vectors that transmit the infection breed in fast flowing rivers, and because the embryonic stages or microfilariae (mf) of the parasite *Onchocerca volvulus* can enter the eye and cause irreversible visual impairment and blindness [1]. This is a protracted and chronic process because continual exposure to many infective vector bites is needed to build up a substantial worm burden and ensuing microfilarial infection (of skin and ocular tissues), and because adult female worms (which produce hundreds to thousands of mf daily) live, on average, for ten years [2]. The adult stages (macrofilariae) reside in worm bundles located subcutaneously (palpable nodules) or deeply in the body, where they produce the mf which migrate to the skin (microfilaridermia) and the eyes [3]. Immunological responses to filarial products [4], either of parasite origin or their endosymbiotic *Wolbachia* bacteria [5], lead to long-standing, non-resolving inflammation associated with chronic onchocerciasis pathology [6]. Skin pathology ranges from troublesome itching to (disfiguring) skin changes, including early-stage reactive lesions, and late-stage depigmentation (leopard skin) and atrophy [7]. Moreover, individuals with high microfilaridermia suffer an increased risk of death [8,9], independent of that related to blindness [10], i.e. sighted individuals are also subject to an excess risk of death.

Currently the predominant strategy for onchocerciasis control in Africa is annual community-directed treatment with ivermectin (CDTI) to all those aged five years and older (excluding pregnant or breastfeeding women in the first week after delivery) [11,12]. Ivermectin is a safe and potent microfilaricide, also temporarily reducing the production of live mf by adult female worms for some months after treatment (anti-fertility effect) [13,14]. Spurred by success in some foci of Mali, Nigeria and Senegal [15-17], there has recently been a shift in onchocerciasis control policy in Africa, changing from elimination of the public health burden of onchocerciasis, to elimination of the infection. The African Programme for Onchocerciasis Control (APOC) has a new goal of eliminating onchocerciasis where possible by 2025 [18], and the 2012 London Declaration on Neglected Tropical Diseases (LDNTD) joined the World Health Organization’s (WHO) 2020 Roadmap on NTDs [19] and set goals for elimination of onchocerciasis in selected countries of Africa by 2020 [20]. Rigorous evaluation of the feasibility of achieving these targets, and of the benefits already accrued necessitates the contribution of dynamic models of onchocerciasis infection and disease.

In this paper, a mathematical model of the dynamics of onchocercal disease is developed by linking documented associations between infection, morbidity and mortality to output from our onchocerciasis transmission model (EpiOncho) [14,21-24]. We assess the long-term impact of annual mass drug administration (MDA) of ivermectin on disease and infection in different epidemiological and programmatic settings in savannah areas of Africa. Noteworthy novel features of EpiOncho, overlooked in other modelling studies [25,26], include: (a) a direct association between the intensity of infection with *O. volvulus* mf and excess human mortality [8,9], which was not included in recent estimates of the global burden of onchocerciasis [27], and (b) due consideration of uncertainty in the long-term antifilarial effects of repeated treatments with ivermectin [23].

The stochastic microsimulation ONCHOSIM model has been used to assess the health impact of APOC (as a whole) [26] and the hypothetical feasibility of onchocerciasis elimination in different settings [28]. Based on Plaisier *et al.*[29], ONCHOSIM projections have assumed that ivermecitn has a large cumulative impact on female adult worm fertility (a large anti-macrofilarial action). However, several studies have indicated that this may not be the case [30,31]. Consequently, ivermectin’s long-term impact may currently be overestimated [23]. Finally, there is an increased recognition of the need to inform control programmes with more than one modelling approach in order to enhance the potential of modelling for decision making in public health [32].

## Methods

### Onchocerciasis transmission model

The analysis is underpinned by a deterministic onchocerciasis transmission model (EpiOncho) which describes sex-specific rates of change with respect to time and host age in the mean number of fertile and non-fertile female adult worms per host, the mean number of mf per milligram (mg) of skin, and the mean number of L3 larvae per fly. The model has been refined from the original framework developed by Basáñez and Boussinesq [21], to include age and sex structure of the host population (in particular, age- and sex-dependent exposure to blackfly bites, parameterized using intensity data on microfilaridermia) [22]; the population-level effects of a single [14,24] and multiple [23] treatments with ivermectin, and increased programmatic realism related to patterns of treatment coverage and systematic non-compliance (whose effects can be explored separately) [23]. The assumed human age- and sex-structure of the population reflects demographic characteristics in savannah areas of northern Cameroon [22,33,34], where the prevailing *O. volvulus–Simulium damnosum* sensu *lato* (s.l.) combinations (i.e. savannah parasites–*S. damnosum* sensu stricto (s. str.)/*S. sirbanum*) are responsible for the most severe sequelae of onchocerciasis [1,3]. We assumed a stationary age distribution and a stable (closed) population. The model can reflect pre-control infection levels in a range of hypo- (>35% microfilarial prevalence), meso- (35–60% microfilarial prevalence), and hyperendemic (>60% microfilarial prevalence) onchocerciasis foci [35] by varying the annual biting rate (ABR) of the simuliid vectors (Table 1).

**Table 1:** Summary of baseline (pre-control) modelled epidemiological scenarios

**Pre-control endemicity**	**Annual biting rate (ABR)**^**§†**^	**Annual transmission potential (ATP)**^**¶†**^	**Microfilarial prevalence in all ages**	**Microfilarial prevalence in those aged ≥ 5 years**^**‡**^	Mean microfilarial intensity*in all ages (mf/mg)	Mean microfilarial intensity*in those ≥ **20 yr (mf/mg)**
Mesoendemic	7,300	88	40%	47%	11.2	18.7
Hyperendemic	15,470	373	60%	67%	23.9	40.0
Highly hyperendemic	85,800	4,290	80%	84%	58.9	98.0

^§^Modelled annual biting rate (ABR): the average number of (*S. damnosum* s.str./*S. sirbanum*) bites to which a person is exposed during a whole year.

^¶^Modelled annual transmission potential (ATP): the average number of infective larvae (L3) of *O. volvulus* potentially received during a whole year by a person exposed to the annual biting rate; model assumes perennial transmission.

^†^Both the ABR and ATP are for a proportion of vector blood meals of human origin equal to 0.3 [21].

^‡^Nodule prevalence (recorded by APOC) is converted to microfilarial load in this age range [36].

*Arithmetic mean microfilarial load per mg of skin; note that this is different to the community microfilarial load (CMFL), which is the geometric mean microfilarial load per skin snip in those aged 20 years and above) [37].

#### Ivermectin effects

The model has been modified to incorporate the temporal dynamics of the microfilaricidal and anti-fertility (embryostatic) effects of ivermectin [14,24] (Table 2). Although the initial clinical trial studies that investigated the effects of a single standard dose (150 μg/kg) of ivermectin have shown no evidence of a macrofilaricidal action [38,39], multiple doses of ivermectin over several years may have a cumulative adverse effect on the fertility and/or longevity of adult worms [17,29,40-42]. To account for this potential anti-macrofilarial effect of long-term ivermectin MDA, it was assumed that each dose of ivermectin causes a 7% cumulative reduction in the per capita rate of microfilarial production by adult female worms.

**Table 2:** Effects of ivermectin on various parasite stages

**Parameters**	**Definition**
**Microfilaricidal effect**	The increase in microfilarial mortality.
**Anti-fertility effect**	The temporary reduction in the production of live microfilariae by adult female worms (also known as embryostatic effect).
**Anti-macrofilarial effect**	The long-term, cumulative adverse effect on adult worms due to prolonged exposure of the parasites to the drug (represented in the model as a per dose reduction in the per capita rate of microfilarial production by adult female worms).

This value was motivated by matching EpiOncho’s model output (via varying the per dose reduction) to data on microfilarial load after three years of three-monthly ivermectin treatments (over twelve treatments rounds) presented in Gardon *et al.*[43].

These authors estimated the magnitude and statistical significance of the ivermectin effect on female worm fertility to be greater than on worm mortality; therefore, we chose the former to represent a cumulative, per dose, anti-macrofilarial action of the drug [43]. Despite the higher treatment frequency examined (three-monthly), this dataset was chosen to assess the per dose anti-macrofilarial action of ivermectin, because of the number of treatment rounds the participants were exposed to (over twelve treatment rounds) and because the microfilarial load was presented per mg of skin and not per skin snip (allowing for accurate comparison to EpiOncho’s output).

There is a lack of well-characterized long-term (individual) longitudinal data (including previous treatment history) to estimate more accurately the potential anti-macrofilarial action of ivermectin [23]. The estimated per dose reduction of 7% is consistent with data from recent epidemiological evaluations conducted in areas of Cameroon that have received 13 years of ivermectin distribution. These data do not support the operation of a strong cumulative effect of repeated treatments on the microfilarial production of female worms [31]. In addition, a modelling study by Bottomley *et al.*[30], indicated that ivermectin did not seem to have a cumulative effect on microfilarial production after two and a half years of six-monthly treatments [44]. However, a relatively small reduction would have had a minor initial impact, and thus may not have been detectable in this short time frame.

Our estimated, per ivermectin dose, reduction in the rate of microfilarial production by female worms is smaller than the 30–35% irreversible reduction proposed by Plaisier *et al.*[29], and which these authors estimated by fitting a model to data on five consecutive annual treatments presented in [45], and used in ONCHOSIM [25,26,28]. Therefore, we varied the strength of this anti-macrofilarial action of ivermectin in our sensitivity analysis.

### Estimates of disease burden

**Figure 1:**
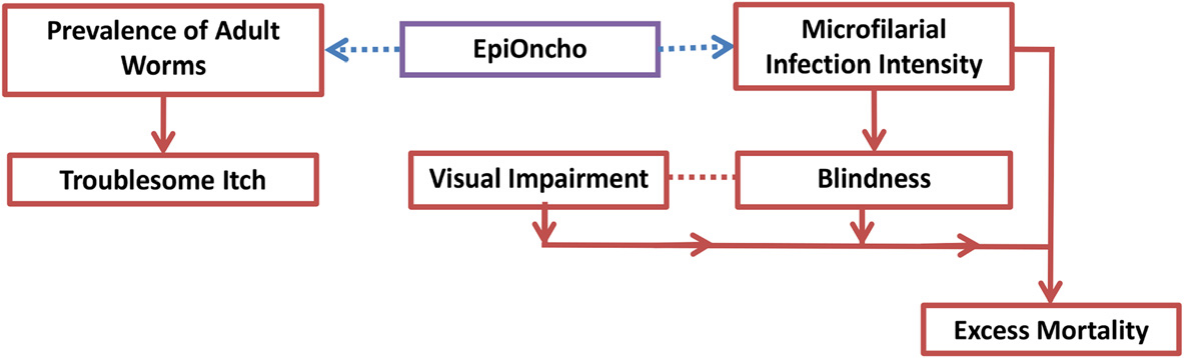
**Schematic representation of the disease model.** Prevalence of troublesome itch was estimated based on a relationship with the prevalence of adult female worms, previously derived using the ONCHOSIM model [25,26]. Incidence of blindness was estimated as a function of microfilarial load (lagged by two years) based on a log-linear Poisson model [46]. The number of individuals with visual impairment was estimated using a published ratio between the prevalence of visual impairment and that of blindness [47]. Excess mortality due to onchocerciasis was assumed to occur via mortality among individuals suffering onchocerciasis-related vision loss (blindness and visual impairment) [10,48], plus an independent (from the former) risk of mortality among sighted individuals with high microfilarial load (lagged by two years) [8,9]. Further descriptions are provided in the main text and Additional file 1: Text S1.

An onchocerciasis disease model was developed by linking output from our dynamic transmission model to the prevalence and incidence of onchocerciasis-associated morbidity and mortality (Figure 1). A summary of how each disease state was represented is found below. Full mathematical details are provided in Additional file 1: Text S1 and Additional file 1: Figures S1-S3. Table S1 summarises the definition and values of parameters and variables for the onchocerciasis disease model.

#### Vision loss

The number of people blind due to onchocerciasis (defined as corrected visual acuity of <3/60 or restriction of visual field to less than 10° in the better eye [47]), was calculated by means of a partial differential equation comprising two rates: the incidence of new onchocercal-related blindness cases, and the loss of already blind individuals due to (excess) mortality [10,48] (see section *Excess Mortality*). The former incidence rate was estimated based on a log-linear Poisson model developed by Little *et al.*[46], which describes incidence of blindness as a function of microfilarial load lagged by two years (fitted to the cohort dataset of the Onchocerciasis Control Programme in West Africa, OCP). The two-year lag provided, which was the best fit to the data [46], reflects that loss of visual acuity is associated with past microfilarial load. Consequently, the decline in prevalence of vision loss was also lagged by two years after the start of ivermectin distribution. The number of individuals with visual impairment or low vision (defined as corrected visual acuity of <18/60 and ≥3/60 in the better eye [47]) caused by onchocerciasis was estimated using a published ratio of 1.78 visual impairment to blindness [47]. Prevalent blindness and visual impairment cases were assumed to be irreversible conditions unresponsive to ivermectin treatment [49], which does not reverse established ocular sequelae (also including sclerosing keratitis and optic nerve atrophy).

#### Troublesome itch

Troublesome itch is thought to be associated with the presence of infection [50] but not with microfilarial infection intensity [51,52]. Hence, we applied a relationship between the prevalence of troublesome itch and of adult female worms, previously derived using ONCHOSIM [25,26]. Troublesome itch was related to the presence of female adult worms because the association between the presence of mf and troublesome itch does not hold during ivermectin treatment, the reduction in prevalence of itch being smaller and more delayed than the drop in microfilarial prevalence and load [25,26,53]. This relationship is subject to considerable uncertainty and so it was varied in the sensitivity analysis (Table 3). In addition, we parameterised the empirical therapeutic effect of ivermectin on troublesome itch using results from a multi-centre trial of ivermectin for treating onchocercal skin disease and severe itching [53] as described in Additional file 1: Text S1. Consequently, there is an initial sharp decline in the prevalence of troublesome itch (as a result of ivermectin’s therapeutic effect) followed by a more gradual decrease as the prevalence of adult worms declines, with a delay driven by the assumed two year pre-patent period [22,54].

**Table 3:** Definitions and values of the parameters explored in the sensitivity analysis

**Parameters**	**Values**
*Initial endemicity:* pre-control (baseline) microfilarial prevalence in the overall population (all ages), as percentage	40% (mesoendemic), 60% (hyperendemic), 80% (highly hyperendemic)
*Therapeutic coverage:* overall proportion of the total population receiving ivermectin at each round, as percentage	60% (moderate) and 80% (high)
*Proportion of systematic non-compliers:* fraction of the eligible population who never take treatment, as percentage	0.1% (lower) and 5% (higher) treatment adherence
*Anti-macrofilarial action of ivermectin:* per dose, cumulative reduction in microfilarial production by ivermectin-exposed female adult worms [23,29], as percentage	7% (small) and 30% (large) anti-macrofilarial effect
*Relationship between infection and troublesome itch:* nonlinear regression coefficient of the association between prevalence of adult female worms and that of troublesome itch [26] (see also eqn. S.16 of Additional file 1)	*α*_2_ ± 25% (stronger or weaker association between prevalence of female worms and prevalence of troublesome itch

#### Excess mortality

Excess mortality due to onchocerciasis was assumed to occur via two independent processes: (a) an additional risk of mortality among individuals suffering onchocercal related vision loss [10,48], and (b) an additional risk of mortality among (sighted) individuals with high microfilarial loads [8,9]. The former (a) was modelled using a risk of mortality among blind and visually impaired individuals that is, respectively, 2.5 and 1.5 times higher than that of fully sighted individuals [48]. The latter (b) was modelled using a published non-linear, host age-dependent association between the relative risk of mortality of sighted individuals and their microfilarial load (lagged by two years) estimated from the OCP cohort dataset [9].

#### Disability-adjusted life years

Disability-adjusted life years (DALYs) due to onchocerciasis were used to combine into a single metric the burden of blindness, visual impairment, troublesome itching (years lived with disability, YLD), and premature death (years of life lost, YLL). The DALYs were estimated using the disability weights provided by the Global Burden of Disease (2004) study [55] (see Additional file 1: Text S2). The YLLs were discounted at a rate of 3% per year, in agreement with WHO guidelines [56]. Further description of the DALY calculations is provided in Additional file 1: Text S2 and Additional file 2: Table S2.

### Model outputs and sensitivity analysis

We estimated the pre-control disease burden associated with onchocerciasis in African savannah areas within the range of endemicities explored (Tables 1 and 3). In addition, the model was used to estimate the overall microfilarial prevalence (all ages) and intensity (reported as the mean microfilarial load per mg of skin in those aged ≥20 years), as this is the age range used for assessment of community microfilarial load (CMFL) [37]) and its associated morbidity and mortality over the course of 15 annual ivermectin treatment rounds. We chose 15 years because (a) it is a reasonable duration for modelling the long-term impact of ivermectin treatment and making comparisons between different scenarios, and (b) epidemiological studies have documented apparent elimination in this approximate timescale [15-17].

The sensitivity of model projections was explored with regards to a range of epidemiological (endemicity setting) and treatment effectiveness (programmatic variables and treatment efficacy) assumptions. Table 3 presents the definitions and values of the parameters that were explored in the sensitivity analysis.

## Results

### Pre-control disease burden

**Figure 2:**
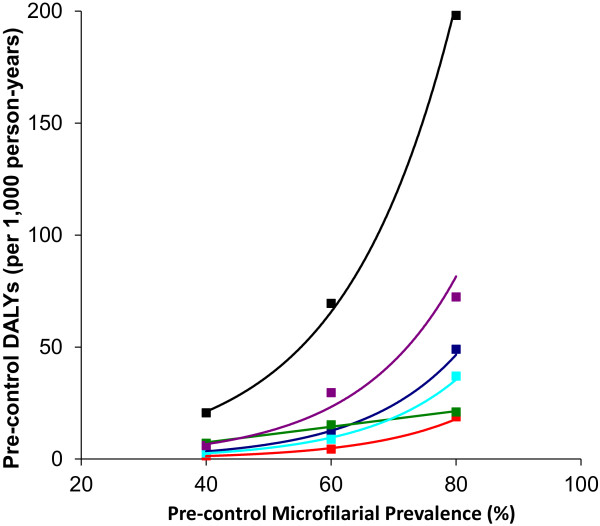
**Relationship between the level of endemicity and pre-control disease burden associated with onchocerciasis in savannah areas of Africa.** Total disability adjusted life-years (DALY) associated with onchocerciasis (black); years of life with disability (YLD) associated with onchocerciasis-related blindness (dark blue); YLD associated with onchocerciasis-related visual impairment (red); YLD associated with onchocerciasis-related troublesome itch (green); years of life lost (YLL) associated with vision loss (light blue); YLL associated with high microfilarial load (purple).

**Figure 3:**
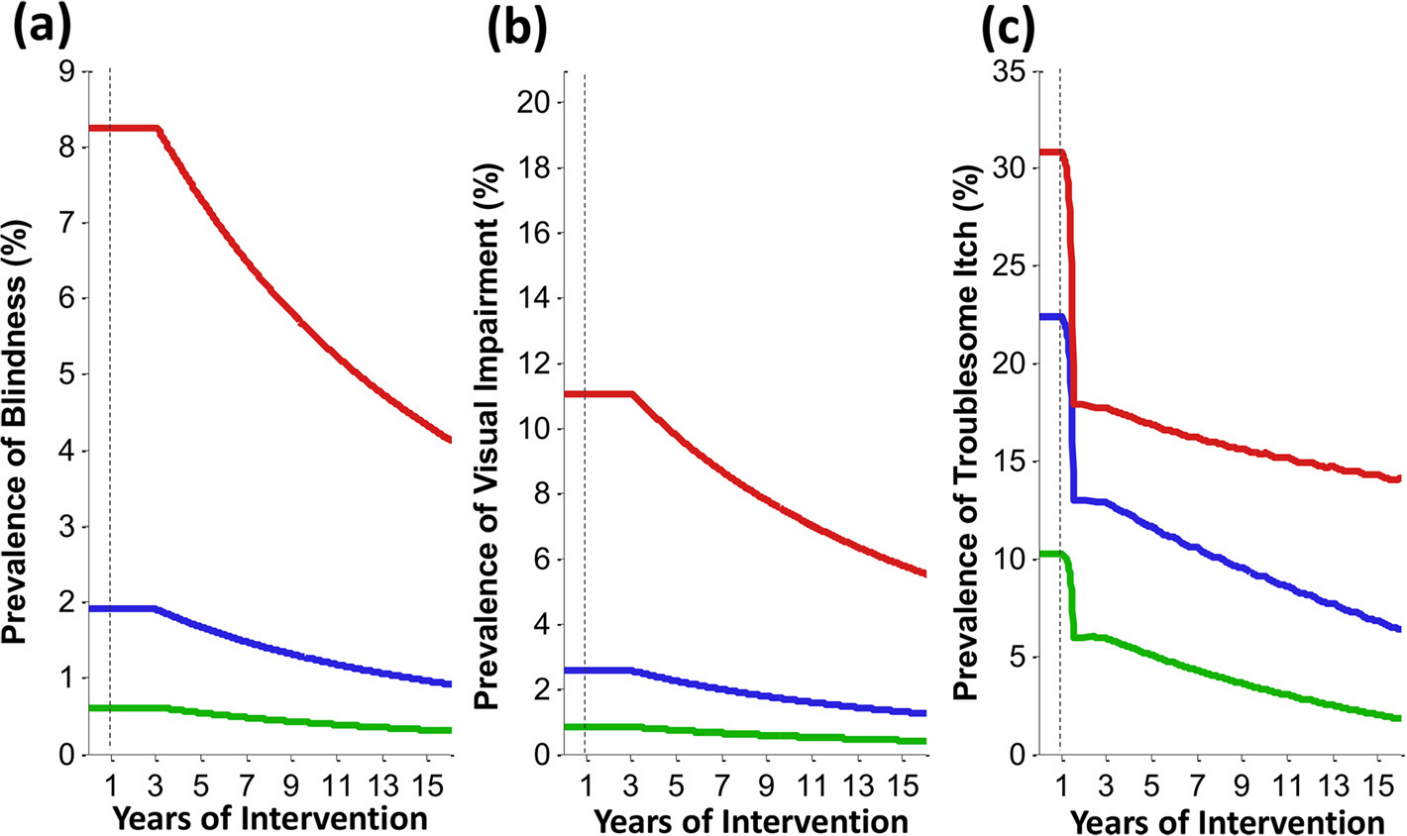
**Impact of annual ivermectin distribution on the morbidity associated with onchocerciasis in savannah areas of Africa. (a)** Prevalence of blindness due to onchocerciasis (across all ages). **(b)** Prevalence of visual impairment due to onchocerciasis (across all ages). **(c)** Prevalence of troublesome itch due to onchocerciasis (across all ages). Red, blue and green lines correspond, respectively, to a baseline endemicity of 80%, 60% and 40% microfilarial prevalence. Results shown assume a therapeutic coverage of 80%, 0.1% of systematic non-compliance, perennial transmission, and a 7% cumulative reduction in microfilarial production by female adult worms per ivermectin dose. The commencement of the intervention at year 1 is represented by the vertical dashed lines. Delays in the decrease of blindness and visual impairment are due to a two-year lag between vision loss in the present and microfilarial infection in the past. The initial sharp decline in troublesome itch is due to the assumed therapeutic effect of ivermectin followed by a more gradual decrease as adult worm prevalence declines.

Before the inception of mass ivermectin distribution and in the absence of other control interventions, infection by *O. volvulus* in African savannah areas can be associated with a large burden of disease, which is non-linearly related to baseline endemicity. This is illustrated by the pre-control (total) DALY burden stratified by baseline endemicity in Table 4 and Figure 2. Relative to the burden for the mesoendemic level (represented by a microfilarial prevalence of 40%), the burden corresponding to the hyperendemic level is three times as high, and for the highly hyperendemic level (80% microfilarial prevalence at baseline), is seven times as high. In terms of the specific pre-control burdens of morbidity and mortality, onchocerciasis was associated with: (a) high levels of blindness and visual impairment, with the baseline overall prevalence (across all ages) of onchocercal related blindness reaching over 8% in highly hyperendemic areas (Figures 3a and 3b); (b) high levels of troublesome itch (Figure 3c), with the estimated pre-control overall prevalence reaching over 30% in highly hyperendemic areas, and (c) a substantial incidence of excess mortality (Table 4 and Figure 2). The YLLs associated with high microfilarial load were responsible for a substantially higher proportion of excess host mortality than those associated with onchocercal related vision loss (Table 4 and Figure 2).

**Table 4:** Baseline (pre-control) model-derived burden of disease (DALYs) associated with onchocerciasis in savannah areas of Africa at different levels of endemicity

	**Disability Adjusted Life Years (DALYs)**^**§**^**(per 1000 person-years)**					
	**Years of life with disability (YLD)**			**Years of life lost (YLL)**^**†**^		
**Pre-control endemicity**‡	Blindness (ratio)*	Visual impairment (ratio)*	Troublesome itch (ratio)*	Associated with vision loss (ratio)*	Associated with high microfilarial load (ratio)*	Total DALY burden (ratio)*
Mesoendemic	3.6	1.4	7.0	2.7	5.9	20.6
Hyperendemic	11.4 (3.2)	4.4 (3.1)	15.3 (2.2)	8.8 (3.3)	29.6 (5.0)	69.5 (3.4)
Highly hyperendemic	49.0 (13.6)	18.8 (13.4)	21.0 (3.0)	37.0 (13.7)	72.3 (12.3)	198.7 (9.6)

^§^See Additional file 1: Text S2 for a detailed description of the methods used to calculate DALYs.

^†^In line with WHO guidelines, a discount rate of 3% was applied to YLLs [56].

*Ratio of metric with respect to the value for the mesoendemic level.

‡ Pre-control microfilarial prevalence as in Table 1.

### Impact of ivermectin on microfilarial prevalence and intensity

**Figure 4:**
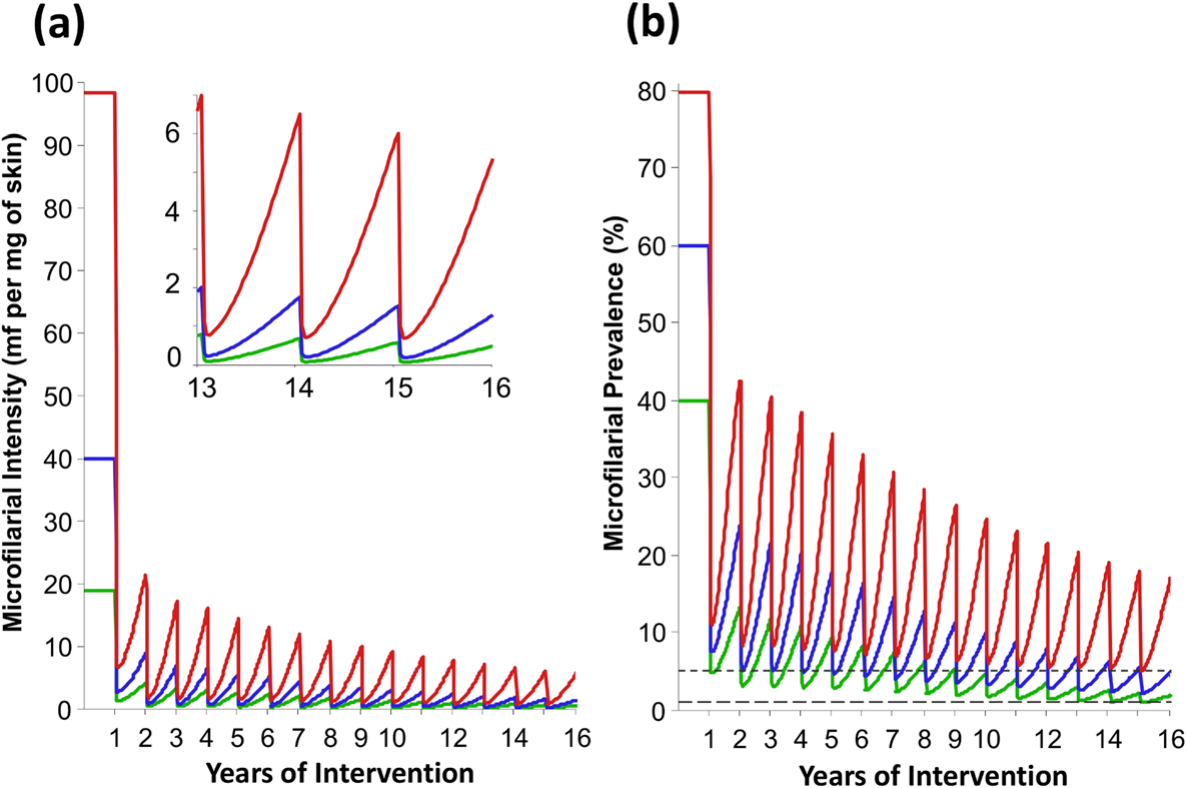
**Impact of annual ivermectin distribution on the intensity (a) and prevalence (b) of microfilarial infection.** Red, blue and green lines correspond, respectively, to a baseline endemicity of 80%, 60% and 40% microfilarial prevalence. Microfilarial intensity is quantified as the mean microfilarial load per mg of skin in those aged ≥ 20 years. The dashed horizontal lines illustrate the upper and lower bounds (5% and 1% prevalence) of the current operational thresholds for cessation of treatment, namely an observed microfilarial prevalence below 5% in all surveyed villages and 1% in 90% of the surveyed villages [57]. Assumptions are as in legend of Figure 3. The inset in Figure 4 **(a)** zooms in microfilarial infection intensity (in the ≥ 20 yr of age) for the last four years of the simulated intervention programme.

Long-term (15 years of consecutive) annual ivermectin distribution is projected to reduce progressively and markedly (by more than 90%), the intensity of microfilarial infection (measured in the population aged ≥20 years). However, due to the dynamic nature of ivermectin’s action on the production of mf by adult female worms, these parasite stages reappear in the skin (with the potential of being transmitted to vectors) between consecutive annual treatments (Figure 4a). The degree of skin repopulation by mf is strongly related to pre-control endemicity (reflecting adult worm burden and vector density) and is substantially larger for (highly) hyperendemic areas. The impact on microfilarial prevalence (all ages) is less marked (yet larger than approximately an 80% reduction) than that on microfilarial intensity (due to the nature of the non-linear relationship between these two variables, Additional file 1: Figure S1) and decreases with increasing levels of pre-control endemicity (Figure 4b).

### Impact of ivermectin on Onchocerciasis disease burden

#### Morbidity

**Figure 5:**
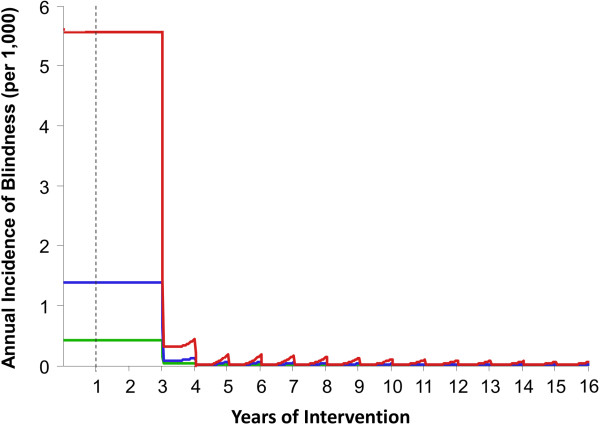
**Impact of annual ivermectin distribution on incidence of blindness due to onchocerciasis in savannah areas of Africa.** Red, blue and green lines correspond to, respectively, a baseline endemicity of 80%, 60% and 40% microfilarial prevalence. The commencement of the intervention at year 1 is represented by the vertical dashed line. The initially delayed decrease is due to a two-year lag between blindness incidence in the present and microfilarial load in the past. Results shown assume a therapeutic coverage of 80%, 0.1% of systematic non-compliance, perennial transmission, and a 7% cumulative reduction in microfilarial production by female adult worms per ivermectin dose.

Model outputs indicate that long-term annual distribution of ivermectin has an enormous impact on the morbidity associated with onchocerciasis (Figure 3). Two years after the start of ivermectin distribution, the incidence of blindness (associated with lagged microfilarial load) is projected to fall to very low levels (Figure 5). By contrast, the proportion of individuals with blindness and visual impairment due to onchocerciasis declines more gradually, as prevalent cases are slowly removed due to host mortality, but not replaced at the same pre-control incidence level. There is a very strong initial decline in the prevalence of troublesome itch due to the therapeutic benefit of ivermectin on cutaneous pathologies [53], followed by a more steady decline during the programme due to a gradual reduction in transmission (and prevalence of adult female worms), the magnitude of which depends on pre-control endemicity level (the higher the level the lower the rate of decrease). However, there is considerable uncertainty regarding the impact of ivermectin on troublesome itching (Additional file 1: Figure S4).

#### Excess mortality

**Figure 6:**
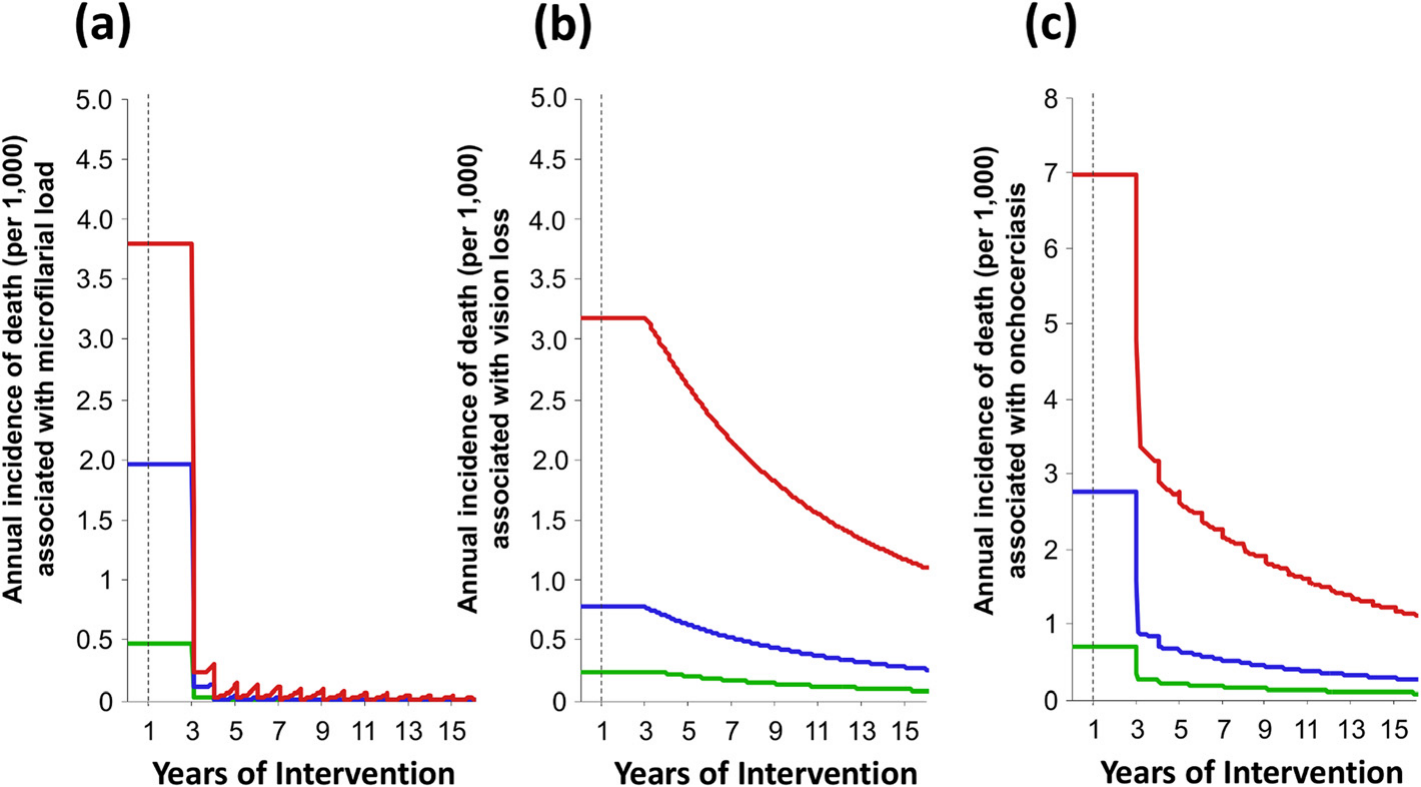
**Impact of annual ivermectin distribution on the excess mortality associated with onchocerciasis in savannah areas of Africa*. *(a)** Associated with a high microfilarial load. **(b)** Associated with vision loss (blindness/visual impairment). **(c)** Total excess death associated with onchocerciasis. Red, blue and green lines correspond, respectively, to a baseline endemicity of 80%, 60% and 40% microfilarial prevalence. Assumptions are as in Figure 3. The commencement of the intervention at year 1 is represented by the vertical dashed lines. The initially delayed decrease of excess mortality is due to a two-year lag between incidence of death in the present and microfilarial load in the past.

Under ivermectin distribution the incidence of excess mortality associated with high microfilarial load is projected to decrease rapidly to low levels (Figure 6a). The decline is delayed by two years after the start of ivermectin distribution because the incidence of excess mortality due to infection is associated with microfilarial load experienced two years in the past [8]. The incidence of excess mortality associated with onchocercal related vision loss decreases at a slower rate, following the decline in the prevalence of vision loss (Figure 6b).

#### Disability-adjusted life years

The overall impact (on morbidity and mortality) of ivermectin distribution on the DALY disease burden associated with onchocerciasis is illustrated in Additional file 1: Figure S5.

### Impact of programmatic variables: therapeutic coverage and compliance patterns

Varying the level of therapeutic coverage and the proportion of systematic non-compliers influences the projected impact of long-term ivermectin distribution on microfilarial prevalence and intensity. An increased level of overall therapeutic coverage (from 60% to 80%), or an increased level of treatment adherence (a decreased proportion of systematic non-compliers, from 5% to 0.1%) decreases microfilarial prevalence and intensity measured 1 year after the 15^th^ annual treatment (Tables 5 and 6). The proportional reduction in infection due to improved coverage or compliance is generally greater for the meso- and hyperendemic levels than for the highly hyperendemic level. By contrast, the proportional reductions in onchocerciasis-associated disease burden resulting from improved coverage and compliance were relatively small in comparison.

**Table 5:** The effect of annual ivermectin treatment coverage on the microfilarial prevalence and intensity of onchocerciasis infection and its associated morbidity and mortality according to baseline endemicity

**Pre-control endemicity‡**	**Mesoendemic**			**Hyperendemic**			**Highly hyperendemic**		
**Therapeutic coverage**	**60%**	**80%**	**%**^**†**^**change**	**60%**	**80%**	**%**^**†**^**change**	**60%**	**80%**	**%**^**†**^**change**
Skin microfilarial prevalence^§^ (%)	3.46	1.84	47%	9.52	4.74	50%	27.53	16.69	39%
Microfilarial intensity^§^ (mf/mg)	1.08	0.49	55%	3.1	1.31	58%	11.14	5.47	51%
Blindness prevalence^§^ (%)	0.299	0.297	0.67%	0.95	0.91	4%	4.25	4.13	3%
Visual impairment prevalence^§^ (%)	0.4015	0.4014	0.02%	1.27	1.22	4%	5.7	5.54	3%
Troublesome itch prevalence^§^ (%)	2.79	1.80	36%	9.43	3.73	32%	17.38	14.09	19%
Excess mortality annual incidence^§^ (per 1000)	0.09	0.08	11%	0.29	0.26	10%	1.39	1.13	19%

^§^Values correspond to model outputs 12 months after the 15th annual ivermectin treatment assuming perennial transmission, 0.1% of systematic non-compliance (high treatment adherence) and a 7% cumulative, per ivermectin dose, reduction in the rate of microfilarial production by adult female worms. Microfilarial infection intensity is quantified as arithmetic mean microfilarial load per mg of skin in those aged ≥ 20 years.

^†^Proportional (percent) reduction in parasitological, morbidity and mortality indicators relative to the lower (60%) treatment coverage of the total population (overall therapeutic coverage).

‡ Pre-control microfilarial prevalence as in Table 1.

**Table 6:** The effect of the proportion of systematic non-compliance with annual ivermectin treatment on the microfilarial prevalence and intensity of onchocerciasis infection and its associated morbidity and mortality according to baseline endemicity

**Pre-control endemicity‡**	**Mesoendemic**			**Hyperendemic**			**Highly hyperendemic**		
**Systematic non-compliance**	**5%**	**0.1%**	**%**^**†**^**change**	**5%**	**0.1%**	**%**^**†**^**change**	**5%**	**0.1%**	**%**^**†**^**change**
Skin microfilarial prevalence^§^ (%)	2.6	1.84	29%	6.68	4.74	29%	20.08	16.69	17%
Microfilarial intensity^§^ (mf/mg)	0.8	0.49	39%	2.2	1.31	40%	8.18	5.47	33%
Blindness prevalence^§^ (%)	0.299	0.297	1%	0.95	0.91	4%	4.26	4.13	3%
Visual impairment prevalence^§^ (%)	0.41	0.4	2%	1.27	1.22	4%	5.7	5.54	3%
Troublesome itch prevalence^§^ (%)	2.22	1.80	19%	7.66	3.73	16%	14.95	14.09	6%
Excess mortality annual incidence^§^ (per 1000)	0.09	0.08	11%	0.3	0.26	13%	1.29	1.13	12%

^§^Values correspond to model outputs 12 months after the 15th annual ivermectin treatment assuming perennial transmission, an overall treatment coverage of 80% (high coverage), and a 7% cumulative, per ivermectin dose, reduction in the rate of microfilarial production by adult female worms. Microfilarial infection intensity is quantified as arithmetic mean microfilarial load per mg of skin in those aged ≥ 20 years.

^†^Proportional (percent) reduction in parasitological, morbidity and mortality indicators relative to the higher (5%) proportion of systematic non-compliance (lower treatment adherence).

‡ Pre-control microfilarial prevalence as in Table 1.

### Impact of the efficacy of ivermectin anti-macrofilarial action

The magnitude of the assumed anti-macrofilarial effect of ivermectin (i.e. the per dose proportion by which microfilarial production by female worms is cumulatively reduced) influenced the long-term impact of annual ivermectin distribution on microfilarial prevalence and intensity. The higher value (30%, as assumed in ONCHOSIM [25,26,28]) had a more pronounced effect than the lower (7%) value (Additional file 1: Figure S6 in comparison to Figure 4). However, this effect also depended on the baseline level of onchocerciasis endemicity; the lower the pre-control endemicity, the smaller the impact of assuming the stronger anti-macrofilarial effect (Additional file 2: Table S3). By contrast, the magnitude of the anti-macrofilarial effect had little influence on the impact of annual ivermectin MDA on onchocerciasis-associated disease burden (Additional file 2: Table S3).

## Discussion

### The influence of the epidemiological setting

#### Pre-control disease burden

In the absence of control interventions, onchocerciasis poses a high disease burden which is non-linearly related to pre-control endemicity level. Model outputs of baseline prevalence of onchocercal related vision loss and troublesome itch in different epidemiological settings are consistent with published data [50,58-60]. Our estimated blindness rates are in good agreement with those reported prior to the commencement of interventions in the core area of the former OCP [58-60]. However, there is heterogeneity in reports of (observed) prevalence of onchocerciasis-associated morbidity, particularly regarding the prevalence of troublesome itch (Additional file 1: Figure S4) [50,58-60].

The estimated DALYs include the excess mortality of sighted individuals with heavy microfilarial loads [8,9], which has not been considered elsewhere. At baseline, the contribution of this mortality associated with infection was greater than the mortality associated with vision loss, and the difference between these two components of premature death increased with increasing levels of pre-control endemicity. This suggests that premature death related to onchocerciasis, and consequently its overall contribution to disease burden may be higher than previously estimated [25-27,48]. Recent estimates of the global disease burden of onchocerciasis [27], which did not include excess host mortality, are therefore underestimated.

#### Impact of ivermectin on microfilarial prevalence and intensity

The impact of long-term annual ivermectin distribution on onchocerciasis prevalence and intensity decreases with increasing levels of baseline (pre-control) endemicity, consistent with other modelling studies [28,57,61]. Although our projections indicate that prolonged annual ivermectin distribution reduces substantially the ocular morbidity and excess mortality associated with onchocerciasis, partly due to very large reductions in microfilarial infection intensity, its impact on the prevalence of infection (and arguably on transmission) is less pronounced. (This will be the product of a combined effect of the non-linear relationship between microfilarial prevalence and intensity, and the relaxation of the density-dependent processes that affect parasite development and vector survival incorporated in the model [62].) This finding is in accordance with the conclusions of a review assessing the impact of repeated ivermectin MDA in the former OCP area [63] and highlights that although the onchocercal disease burden will be markedly reduced, and likely eliminated as a public health problem, continued drug distribution at high levels of treatment coverage and compliance will be vital to interrupt transmission and eliminate the infection reservoir.

After 15 years of annual ivermectin MDA, with consistently high therapeutic coverage, compliance and drug efficacy, projected values of microfilarial prevalence in mesoendemic (1.8%) and hyperendemic (4.7%) areas (Figure 4b, Table 5), approach the operational thresholds for treatment interruption followed by surveillance (OTTIS) proposed by APOC [57]. (These thresholds are defined by a prevalence less than 5% in all surveyed villages and less than 1% in 90% of these and <0.5 infective larvae per 1,000 flies.) Hence, our results are consistent with epidemiological observations in Mali, Senegal and Nigeria after 15–17 years of ivermectin distribution [15-17].

Projected reductions in microfilarial prevalence and intensity were less optimistic for higher levels of the hyperendemicity range (80% initial microfilarial prevalence). In such settings there would be a higher rate of microfilarial reappearance in the skin between consecutive treatments (as adult female worms resume microfilarial production and transmission continues with high vector densities in the absence of vector control). Although under repeated and prolonged ivermectin treatment this rebound in microfilarial intensity was found not to have severe implications for morbidity, it will make it harder to achieve the proposed OTTIS, so our results advise caution when generalising conclusions regarding the feasibility of parasite elimination with annual ivermectin treatment to areas of high pre-control endemicity and perennial transmission.

The shift in onchocerciasis control policy in Africa, from the elimination of morbidity to the elimination of infection [18], means that the dynamics of transmission during inter-treatment periods is increasingly relevant, highlighting the value of mathematical models in capturing the population dynamic effects of underlying biological and epidemiological processes. In particular, our results indicate that if ivermectin does not have a strong anti-macrofilarial action (a strong action has been assumed in ONCHOSIM [25,26,28]), elimination in highly hyperendemic areas is not feasible with annual ivermectin MDA alone. This conclusion is supported by a range of recent epidemiological reports by Katabarwa and co-workers which provide evidence of continued transmission after more than 15 years of annual ivermectin treatment in foci of Cameroon and Uganda with high pre-control endemicity or transmission levels [64-66]. Our model projections, combined with these epidemiological observations, underscore the importance of developing novel interventions and implementing optimal combinations of currently available tools [67].

#### Impact of ivermectin on Onchocerciasis disease burden

Prolonged annual ivermectin distribution is undoubtedly highly effective at reducing the morbidity and excess mortality associated with onchocerciasis. Our projections of a steady decline in the prevalence of blindness, agree with studies investigating the long-term impact of onchocerciasis control on vision loss as well as with ONCHOSIM projections [26,59,60,68,69]. However, our projected reduction in onchocercal related vision loss was less than that reported by Emukah *et al.*[70], who observed a fall in prevalence from 16% to 1% (a 95% reduction) after only eight years of annual ivermectin distribution. This difference could be explained by a higher incidence of excess mortality experienced by individuals with vision loss in the study area [70] than assumed in our model. Others have assumed that four rounds of ivermectin treatment would reduce the burden of visual impairment and blindness by 35% [71]. In our model there is no therapeutic benefit of ivermectin on (irreversible) vision loss; therefore, reductions in prevalence are due to gradual mortality of those with blindness/visual impairment. This contrasts with the faster reduction in the incidence of blindness, which reaches very low levels within a few years of ivermectin MDA (due to its pronounced effect on microfilarial load). However, onchocerciasis-related vision loss may still account for a non-negligible disease burden during on-going control programmes due to remaining prevalent cases. The contribution of prevalent blindness cases was not included in recent estimates of the global burden of onchocercal disease [27].

Model outputs indicating that the overall prevalence of troublesome itch due to onchocerciasis would roughly halve after 5–6 years of annual ivermectin treatment are consistent with data from a multi-centre trial assessing the impact of CDTI on itching and skin disease within APOC [72]. This study consisted of two cross-sectional surveys using a standardised study protocol across seven sites. Other authors have assumed that four rounds of ivermectin treatment would reduce the prevalence of troublesome itching by 85% [71], but this optimistic expectation is not supported by the results of [72] or our modelling outputs. With the exception of two studies by Whitworth *et al.*[73,74], which concluded that ivermectin had no effect on skin disease, our projected reductions are in broad agreement with the literature [53,72,75-77]. Subsequent studies by Whitworth *et al.* using a longer time period and an improved study design, reported a reduction in troublesome itch of 30% after six years of annual ivermectin treatment [76]. It should be noted that after 15 years of ivermectin treatment in mesoendemic areas, our projections indicate a small (1.8%) residual prevalence of troublesome itch. This is associated with a substantial degree of uncertainty due to limitations of available data and a lack of long-term longitudinal data to parameterise accurately a potential cumulative reduction in itching prevalence.

### The influence of programmatic and drug efficacy variables

#### Therapeutic coverage and compliance patterns

Varying levels of overall coverage (comparing a moderate therapeutic coverage of the total population of 60% with a higher coverage of 80%), and levels of systematic non-compliance (comparing a low treatment adherence, with 5% of individuals never taking treatment with a high compliance of only 0.1%) had little effect on the substantial impact that regular and prolonged ivermectin treatment has on the morbidity and excess mortality associated with onchocerciasis. However, both these programmatic considerations had a marked influence on the projected impact of annual ivermectin treatment on the prevalence and intensity of microfilarial infection [23]. This indicates that under the new impetus for elimination of infection (as opposed to elimination of morbidity only) [18], the proportion of the population that for whatever reason always refuse treatment, cannot take it, or cannot be reached will become very important in terms of achieving parasite elimination goals. Operational research efforts should be made to understand what proportion of the population (stratified by age and sex) do not take treatment [78,79], what are the reasons behind this non-compliance, and how to develop effective strategies to increase treatment adherence [23]. In addition, it will also be important to ascertain whether and to what extent systematic non-compliers are represented in monitoring and evaluation sampling protocols; it is conceivable that individuals who are non-compliant to treatment may not be present during parasitological assessments, biasing results and potentially leading to erroneous decisions concerning cessation of treatment.

#### Anti-macrofilarial effect of ivermectin

Based on [30,31,43], it was assumed that ivermectin would only have a relatively small anti-macrofilarial action, i.e., effecting a 7% cumulative reduction on the rate of microfilarial production by adult female worms per standard dose. Due to uncertainty in the magnitude of this effect [23], analyses were also conducted assuming the operation of a stronger (30% per dose) anti-macrofilarial action (as previously assumed in ONCHOSIM [25,26,28]) [29]. Varying this parameter had a prominent impact on projected microfilarial prevalence and intensity, but did not greatly affect the projected impact on disease burden. The degree to which the magnitude of the anti-macrofilarial effect influenced infection levels decreased with decreasing pre-control endemicity, reflecting the lower degree of residual transmission occurring between consecutive treatments [23].

### Potential limitations

Currently, our transmission and disease EpiOncho model has been calibrated for savannah settings of Africa; thus, results are not necessarily directly generalisable to forest settings which have different relationships between infection and sequelae [1,3], different transmission intensities [80], and where onchocerciasis vectors are different members of the *Simulium damnosum* s.l. complex [81] (but also see [82] for a review of blindness associated with different epidemiological and entomological settings in savannah and forest areas).

The present version of the model assumes a stationary age distribution and a stable (closed) population and consequently does not account for potential effects of onchocerciasis-related excess host mortality on the population distribution. Additionally, the results presented here assume that transmission is perennial (i.e. occurs all year round). Further investigation of the influence of different seasonal transmission patterns on the optimal timing of ivermectin distribution will be essential and is underway.

As in other modelling studies of the health impact of ivermectin [26], we included disease manifestations for which data were available for model parameterisation. However, we have not yet quantified disease burden associated with other types of skin disease (such as leopard skin among others) [50,72], and therefore we may be underestimating the pre-control disease burden and the overall health impact of ivermectin. Furthermore, onchocerciasis is associated with epilepsy [83,84], nodding disease, and Nakalanga syndrome [85,86], which have not yet been included in disease models. It is clear that further work and data are required to improve assessment of the disease burden associated with onchocerciasis in future iterations of the Global Burden of Disease study.

EpiOncho is a deterministic model and does not account for the influence of random events (which become particularly important at low infection levels). Therefore, it cannot be used to investigate formally the probability of reaching elimination, which requires a stochastic model.

Finally, it is noteworthy that most models (including ours) are parameterised with data collected prior to the onset of control interventions, and it is possible that the relationships between infection, transmission and the subsequent development of morbidity could be influenced by the treatment per se [87]. Consequently, any model-derived predictions of the long-term impact of ivermectin on both the dynamics of onchocercal infection and its disease burden (particularly regarding troublesome inching) are somewhat uncertain.

### Programmatic considerations

Onchocerciasis control programmes based on annual ivermectin distribution have been in operation in Africa for a considerable time (since 1988 and early 1990’s in some countries of the OCP, and since 1995 to late 1990’s in countries under the umbrella of APOC). Our results confirm those of other authors [26,63] in concluding that this strategy is highly effective in controlling onchocerciasis-related morbidity and reducing dramatically the incidence of new cases of ocular disease. Our work also reveals, for the first time, that the incidence of excess host mortality associated with heavy microfilarial infection (in sighted individuals) would also plummet after some initial lag. It is, therefore, highly likely that the goals of eliminating the public health burden of onchocerciasis will be met within the timeframes agreed by the international global health community, drug donors, project funders and control programmes. The rates at which morbidity in general, and each disease state in particular, decline in the human population will depend on the epidemiological setting, the initial intensity of infection and transmission, and to a much lesser extent on programmatic variables (although only two, moderate and high, values of therapeutic coverage were investigated here). It is anticipated that much lower levels of therapeutic coverage, poor geographical coverage or interruption of programmes due to conflict, population displacement or weak programme implementation among other factors, would be detrimental to the reaching of morbidity elimination goals. A stumbling block here is the existence of areas coendemic for *Loa loa* infection [88], in which ivermectin treatment may be contraindicated in those with very high loiasis microfilaraemia because of the risk of severe adverse events [89]. This represents a big threat to the possibility of ridding Africa of onchocerciasis.

The influence of epidemiological and programmatic factors is very different regarding the feasibility of achieving infection elimination goals, and here we need to give a more cautionary appraisal. This goal will probably be achievable in mesoendemic, and possibly in the lower end of the hyperendemic spectrum (provided high treatment effectiveness is sustained). However, even under enthusiastic scenarios of uninterrupted annual ivermectin treatment, unwaveringly high therapeutic coverage/compliance, and intact drug efficacy, settings with initially very high infection prevalence will challenge the programmes in their attempt to reach interruption of transmission with annual ivermectin distribution alone. This indicates that (highly) hyperendemic settings will require implementation of innovative approaches or optimised combination of existing ones; for instance, implementation of biannual ivermectin treatment to reduce the amount of remaining transmission between consecutive treatment rounds [90], concomitant vector control where possible [91], and treatment (on a test & treat basis) with macrofilaricidal therapies such as doxycycline, proven to sterilise permanently female parasites and kill adult worms [92].

Furthermore, it must be borne in mind that at present, proposed operational thresholds for tentative cessation of treatment (and initiation of post-control surveillance) are, by and large, empirical, based on the very stages most affected by treatment (and therefore not truly representative of the fate of the parasite population), and prone to decreased sensitivity of current diagnostics. Their relationship with transmission breakpoints (parasite densities below which the worm population would not be able to maintain itself) is largely unknown [61].

## Conclusions

The excess mortality of sighted individuals with heavy microfilarial loads [8,9], which has not been considered elsewhere, contributes to a considerable number of years of life lost in onchocerciasis endemic populations. Consequently, the overall disease burden of onchocerciasis and ivermectin’s impact on health have thus far been underestimated [25-27,48].

Long-term annual ivermectin treatment is highly effective in reducing the morbidity and excess mortality associated with onchocerciasis. Consequently, the goals of eliminating the public health burden of onchocerciasis will most likely be met in those areas where long-term, annual ivermectin distribution is feasible. However, due to the dynamic nature of ivermectin’s action on the production of microfilariae [14], these parasite stages will reappear in the skin between consecutive annual treatments; the degree of microfilarial repopulation is substantially larger in (highly) hyperendemic areas, making the infection much harder to eliminate. This highlights the importance of carefully considering the characteristics of the settings in which epidemiological and modelling studies are conducted before generalising their results to other areas. In particular, our results indicate that caution is advised when generalising the conclusion of the feasibility of elimination (observed in [15-17]) with annual treatment to areas with a higher pre-control endemicity and perennial transmission, and further highlights the need for continued evaluation of the criteria proposed for stopping ivermectin treatment (recognised in [15,16]). This has important implications for both the WHO’s and APOC’s goals to eliminate onchocerciasis in selected countries of Africa by 2020/2025 [18,20].

Within our range of scenarios, the overall therapeutic treatment coverage and level of systematic non-compliance to ivermectin had little effect on the substantial impact that long-term ivermectin has on onchocerciasis disease burden. However, both variables had marked effects regarding reductions in infection prevalence and intensity. This indicates that, now that the aim is elimination of the infection where possible (instead of only reducing disease burden), the proportion of systematic non-compliers (as well as the overall coverage) will become far more influential in terms of overall success in achieving elimination goals. This highlights the need for further investigation and assessment of the determinants of treatment compliance and indicates that feasibility of achieving the new goals will depend on epidemiological and programmatic variables, precluding a one-size-fits-all approach to onchocerciasis elimination in Africa.

## Abbreviations

ABR: Annual biting rate; APOC: African Programme for Onchocerciasis Control; ATP: Annual transmission potential; CDTI: Community-directed treatment with ivermectin; CMFL: Community microfilarial load; DALY: Disability adjusted life-year; MDA: Mass drug administration; mf: Microfilariae; mg: Milligram; NTD: Neglected tropical disease; OCP: Onchocerciasis Control Programme in West Africa; OTTIS: Operational thresholds for treatment interruption followed by surveillance; s.l.: Sensu lato; s.str.: Sensu stricto; YLD: Years lived with disability; YLL: Years of life lost; WHO: World Health Organization.

## Competing interests

The authors declare that they have no competing interests.

## Authors’ contributions

HCT conducted the modelling and drafted the first versions of the manuscript. MW, TSC and MGB advised on the modelling and commented on the manuscript for intellectual input. MW and MGB helped to draft the final version of the manuscript. All authors read and approved the final manuscript.

## Authors’ information

HCT is a post-doctoral researcher at the Department of Infectious Disease Epidemiology, Imperial College London and the London Centre for Neglected Tropical Diseases, working on health economics and mathematical modelling. MW is a post-doctoral researcher in the Helminth Ecology Research Group working on statistical and mathematical modelling. TSC is a mathematical modeller holding a Junior Research Fellowship at Imperial College. MGB holds a Chair in Neglected Tropical Diseases at Imperial College London and heads the Helminth Ecology Research Group.

## Supplementary Material

Additional file 1 **Supporting Information Text.** Description of the Onchocerciasis Disease Model. **Text S1.** Onchocerciasis Disease Model. **Text S2.** Disability Adjusted Life Years. **Figure S1.** Observed and fitted microfilarial prevalence as a function of mean microfilarial load. **Figure S2.** Comparison of onchocercal itching prevalence vs. nodule prevalence without (a) and with (b) the adjustment factor *α*_3_. **Figure S3.** Human host survivorship function. **Figure S4.** Sensitivity of the impact of annual ivermectin distribution on troublesome itch. **Figure S5.** Impact of annual ivermectin distribution on the DALY burden associated with onchocerciasis in savannah areas of Africa. **Figure S6.** Impact of annual ivermectin distribution on: (a) microfilarial intensity and (b) microfilarial prevalence when assuming a stronger anti-macrofilarial action. Click here for file

Additional file 2: Table S1 Definition and values of parameters and variables for the onchocerciasis disease model. **Table S2.** Definition and values of parameters for the disability-adjusted life years estimates. **Table S3.** The effect of the magnitude of the anti-macrofilarial effect of ivermectin on the microfilarial prevalence and intensity of onchocerciasis infection and its associated morbidity and mortality according to baseline endemicity. Click here for file
