# Supplementary material for: Modelling the impact of ivermectin on River Blindness and its burden of morbidity and mortality in African Savannah: EpiOncho projections
Source: Parasit Vectors. 2014 May 26;7:241. doi: 10.1186/1756-3305-7-241 (PMC4037555; doi:10.1186/1756-3305-7-241)
Supplement: Additional file 1 — Supporting Information Text. Description of the Onchocerciasis Disease Model. Text S1. Onchocerciasis Disease Model. Text S2. Disability Adjusted Life Years. Figure S1. Observed and fitted microfilarial prevalence as a function of mean microfilarial load. Figure S2. Comparison of onchocercal itching prevalence vs. nodule prevalence without (a) and with (b) the adjustment factor α3. Figure S3. Human host survivorship function. Figure S4. Sensitivity of the impact of annual ivermectin distribution on troublesome itch. Figure S5. Impact of annual ivermectin distribution on the DALY burden associated with onchocerciasis in savannah areas of Africa. Figure S6. Impact of annual ivermectin distribution on: (a) microfilarial intensity and (b) microfilarial prevalence when assuming a stronger anti-macrofilarial action. [file 1756-3305-7-241-S1.pdf]

Supporting text. Description of the Onchocerciasis Disease Model

This supporting text describes in full detail the onchocerciasis disease model which was developed by coupling output from our onchocerciasis transmission (EpiOncho) model—namely the mean number of microfilariae per mg of skin, the derived prevalence of microfilariae, and the derived prevalence of adult female worms—to the incidence and prevalence of onchocercal morbidity and mortality using statistically documented associations between infection and disease previously published. Details are also given on how disability adjusted life years (DALYs) were calculated using output from the disease model. Definitions and, where appropriate, values of parameters introduced in the text are given in Supporting Table S1 for the onchocerciasis disease model and Supporting Table S2 for the calculation of DALYs.

S1. Onchocerciasis Disease Model

S.1.1. *Microfilarial Prevalence*

The prevalence of skin microfilariae (prevalence of microfilaridermia), $\pi^{M}_{s,d}(t,a)$, at time *t*, in hosts of age, *a*; sex, *s*, and treatment compliance group, *d*, was calculated as a function of microfilarial load per milligram (mf/mg) of skin, $M_{s,d}(t,a)$, determined by the onchocerciasis transmission model,

$$\pi^{M}_{s,d}(t,a) = 1 - \left[1 + \frac{M_{s,d}(t,a)}{k_M\left[M_{s,d}(t,a)\right]} \right]^{-k_M\left[M_{s,d}(t,a)\right]}, \qquad \text{(S.1)}$$

where $k_M[M_{s,d}(t,a)]$ is given by,

$$k_M\left[M_{s,d}(t,a)\right] = \frac{k_0\, M_{s,d}(t,a)}{1 + k_1\, M_{s,d}(t,a)} \ . \qquad \text{(S.2)}$$

Equation (S.1) is derived as 1 minus the probability of having zero microfilariae, assuming a negative binomial distribution of microfilariae among hosts with mean $M_{s,d}(t,a)$ and overdispersion parameter $k_M[M_{s,d}(t,a)]$. The parameters k_0 and k_1 of Equation (S.2) were estimated by fitting Equation (S.1) by maximum likelihood to paired microfilarial prevalence and intensity data from 25 villages in northern Cameroon [1] (Figure S.1, Table S.1). This relationship, previously described in [2], was refitted assuming an average (arithmetic mean) skin snip sample weight of 1.7 mg as opposed to the 2.84 mg used previously and based on [3]. This lower weight was estimated using the mean skin snip weight from samples collected by Collins *et al*. [4], and is in agreement with other studies [5].

S.1.2 *Human population*

To calculate the stratum-specific population size, $P_{s,d}(a)$, the total population size, *P*, was multiplied by the proportion of individuals of sex *s*, q_s; age *a*, $\rho(a)$; and compliance group *d*, η_d,

$$P_{s,d}(a) = q_s \eta_d \rho(a) P \ . \qquad \text{(S.3)}$$

The age and sex distributions of the population, modelled by the function $\rho(a)$ and the parameter q_s respectively, were estimated from demographic data on individuals living in the Vina Valley region of northern Cameroon [6]. The population structure and size were

assumed to be stationary; the latter set to an arbitrary constant large enough to ensure accuracy of numerical integration.

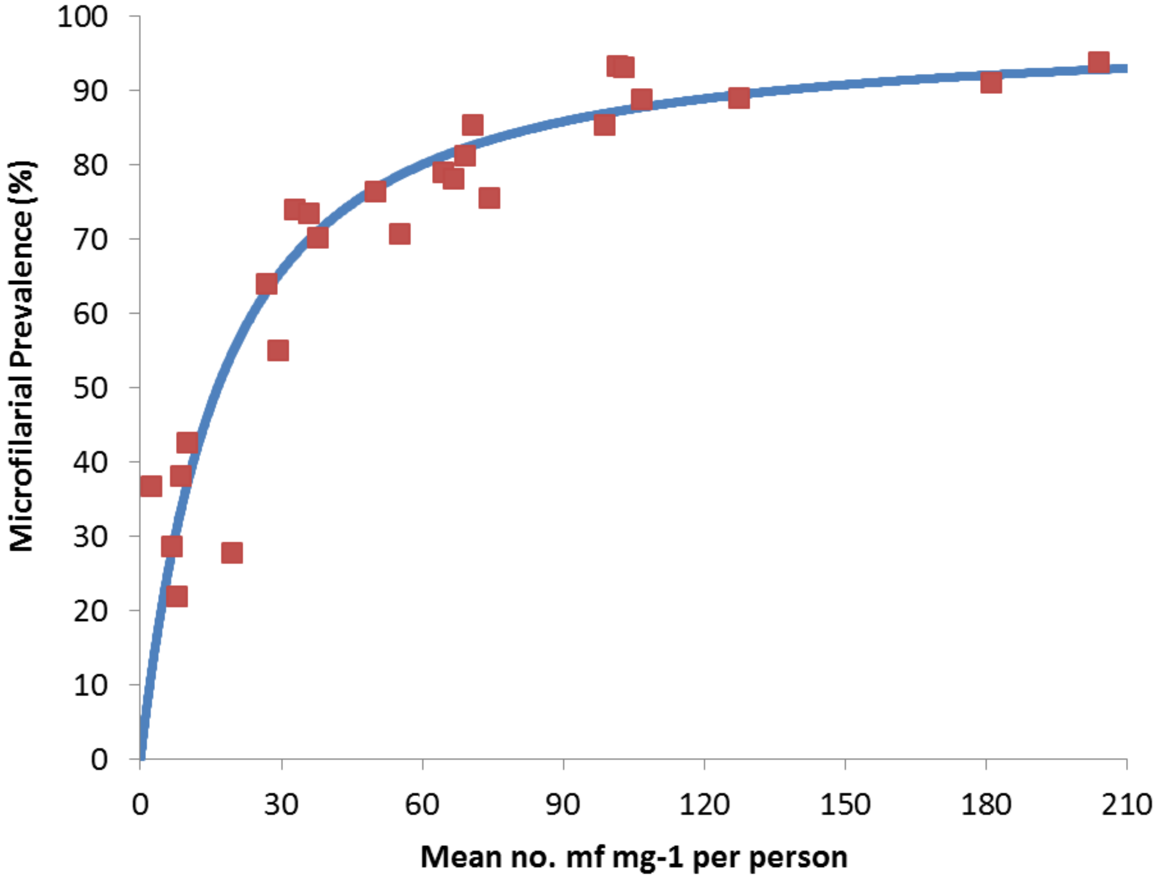

Figure S1. Observed and fitted mcrofilarial prevalence as a function of mean microfilarial load. The data are from 25 north Cameroonian villages studied by Boussinesq (1991) and presented in Basáñez & Boussinesq (1999) [1, 2]. It was assumed that skin snip samples weighed on average 1.7 milligrams [4] (as opposed to the 2.84mg quoted in [3]). Red markers correspond to the age- and sex-adjusted microfilarial prevalence in the communities [2]. The blue line is the function given in equation (S.1) with maximum likelihood estimates k_0 = 0.013 (95% CL 0.011–0.015) and k_1 = 0.025 (95% CL 0.019– 0.033) (Table S1).

S.1.3. *Blindness*

The incidence of blindness due to onchocerciasis, denoted $\nu_{s,d}(t,a)$, is given by the incidence of blindness *not* due to onchocerciasis, $\nu'_{s,d}(t,a)$ (i.e. the background incidence of blindness), subtracted from the *total* incidence of blindness, $\nu^T_{s,d}(t,a)$,

$$\nu_{s,d}(t,a) = \nu^T_{s,d}(t,a) - \nu'_{s,d}(t,a). \tag{S.4}$$

The background incidence of blindness is given by the *per capita* background rate at which individuals of sex *s* and age *a* become blind [7], $\gamma_{0s}(a)$, multiplied by the total number of individuals who are not blind. The latter is given by subtracting the total number of blindness cases in each stratum, $B^T_{s,d}(t,a)$, from the stratum population size, $P_{s,d}(a)$,

$$\nu'_{s,d}(t,a) = \gamma_{0s}(a)\left[P_{s,d}(a) - B^T_{s,d}(t,a)\right]. \tag{S.5}$$

The total incidence of blindness in the population of sex *s* and compliance group *d* is calculated from the background incidence of blindness (Equation (S.5)) multiplied by the relative risk of blindness associated with a mean number of microfilariae per skin snip, $r_{s,d}(t,a)$,

$$\nu^T_{s,d}(t,a) = \nu'_{s,d}(t,a) r_{s,d}(t,a). \tag{S.6}$$

Substituting $\nu'_{s,d}(t,a)$ (Equation (S.5)) and $\nu^T_{s,d}(t,a)$ (Equation (S.6)) into Equation (S.4) yields the desired expression for the incidence of blindness *due to* onchocerciasis,

$$\nu_{s,d}(t,a) = \gamma_{0s}(a)\left[r_{s,d}(t,a) - 1\right]\left[P_{s,d}(a) - B^T_{s,d}(t,a)\right]. \tag{S.7}$$

The relative risk term on the right hand side (RHS) of Equation (S.7) was calculated using a log-linear model previously fitted to data on the incidence of blindness throughout the entire duration of the Onchocerciasis Control Programme in West Africa (OCP) [7],

$$r_{s,d}(t,a) = \exp\left[\gamma_1 M'_{s,d}(t-2,a)\right]\pi^M_{s,d}(t-2,a). \tag{S.8}$$

Here $M'_{s,d}(t-2,a)$ denotes mean microfilarial load per skin snip (as opposed to per mg of skin, see below) lagged by two years, reflecting that loss of visual acuity is associated with past microfilarial load, with the best lag estimated as 2 years [7]. The term γ_1 on the RHS of Equation (S.8) is the regression coefficient for the relative risk of blindness associated with a microfilarial load, as estimated in [7] (Table S.1). The microfilarial load per skin snip was derived by multiplying the microfilarial load per mg of skin (obtained from the transmission model) by the mean weight in milligrams of a skin snip sample taken from the iliac crest using a Holth-type corneoscleral punch. This mean was estimated as 1.7 mg (S.E. = 0.012) from data presented by Collins *et al*. [4]. This conversion was necessary because the OCP did not weigh the skin snips and consequently Equation S.8 was parameterized using data on mf per skin snip rather than per mg. The prevalence term, $\pi^M_{s,d}(t-2,a)$, in Equation (S.8) ensures that the relative risk of blindness *due to* onchocerciasis applies only to those who are infected.

The total number of people blind—the $B^T_{s,d}(t,a)$ term on the RHS of Equation (S.7) —was calculated by means of a partial differential equation comprising two (host age- and sex-dependent) rates: the total incidence of new blindness cases, $v^T_{s,d}(t,a)$ (Equation (S.6)), minus the loss due to mortality of already blinded individuals, with *per capita* rate as the product of the background mortality rate $\xi(a)$ and the excess risk of mortality associated with blindness E^B [8],

$$\frac{\partial B^{T}_{s,d}(t,a)}{\partial t}+\frac{\partial B^{T}_{s,d}(t,a)}{\partial a}=v^{T}_{s,d}(t,a)-B^{T}_{s,d}(t,a)\xi(a)E^{B}. \qquad \text{(S.9)}$$

The expression for the background mortality rate, $\xi(a)$, is given in section 1.6 *Excess Mortality*. The number of blindness cases *due* to onchocerciasis, $B_{s,d}(t,a)$ (as opposed to the *total* number of blindness cases) was calculated in a similar fashion but using $v_{s,d}(t,a)$; the incidence of blindness *due to* onchocerciasis (Equation (S.7)),

$$\frac{\partial B_{s,d}(t,a)}{\partial t}+\frac{\partial B_{s,d}(t,a)}{\partial a}=v_{s,d}(t,a)-B_{s,d}(t,a)\xi(a)E^{B}. \qquad \text{(S.10)}$$

The relative risk of blindness due to onchocerciasis, $r_{s,d}(t,a)$ (Equation (S.8)) was determined by analysing data where blindness was defined as a visual acuity of less than 3/60 based on tests for central vision [7]. It does not include individuals with visual acuity equal to or better than 3/60 but who have a restriction of visual field to less than 10° of fixation. Such individuals would have been classified as blind if peripheral visual field assessment had been conducted. It has been estimated that this approach misses 25% of functional blindness cases [9, 10]. To account for this, the number of age- and sex-dependent blindness cases due to onchocerciasis per stratum (given by the solution of Equation (S.10)) was multiplied by 4/3 [9, 11]. The total number of blindness cases due to onchocerciasis was calculated by integrating over host age and summing over sex and treatment compliance strata,

$$B(t)=\frac{4}{3}\sum_{s}\sum_{d}\int_{a}B_{s,d}(t,a)\,da\,. \qquad \text{(S.11)}$$

The prevalence of onchocercal blindness, $\pi^{B}(t)$, is calculated by dividing the total number of blindness cases by the population size, P,

$$\pi^{B}(t) = \frac{B(t)}{P}. \qquad \text{(S.12)}$$

S.1.4 *Visual Impairment*

The number of visual impairment cases, $V_{s,d}(t,a)$, was calculated using a ratio of 1.78 cases of visual impairment for every case of blindness (when blindness is defined as a visual acuity of less than 3/60 alone) [9]. This ratio was estimated from data collected in hyperendemic onchocerciasis foci (without adjusting for the functionally blind cases as described above), but it was assumed to hold for lower endemicity foci [9]. Hence,

$$V_{s,d}(t,a) = 1.78 \times B_{s,d}(t,a). \qquad \text{(S.13)}$$

The prevalence of onchocercal visual impairment, $\pi^{V}(t)$, is then given by,

$$\pi^{V}(t) = \frac{V(t)}{P}, \qquad \text{(S.14)}$$

where,

$$V(t) = \sum_{s}\sum_{d}\int_{a} V_{s,d}(t,a)\,da. \qquad \text{(S.15)}$$

1.4.1 *The Therapeutic Effect of Ivermectin on Visual Loss*

It was assumed that both blindness and visual impairment are irreversible conditions which do not respond to ivermectin treatment. This is supported by a Cochrane review of placebo-controlled trials that found no statistically significant effect of ivermectin on prevalent vision loss [12].

S.1.5 *Troublesome Itch*

Following [11, 13] the *baseline* prevalence of troublesome itch, $\pi'^{T}_{s,d}(t),$ is defined as the prevalence of troublesome itch in the absence of ivermectin treatment. This is calculated using a previously published association between troublesome itch and the prevalence of adult female worms, $\pi^{W}_{s,d}(t)$ [11, 13],

$$\pi'^{T}_{s,d}(t) = \alpha_1 \pi^{W}_{s,d}(t) + \alpha_2 \left\{1 - \exp\left[\left(\pi^{W}_{s,d}(t)\right)^2\right]\right\} \alpha_3[\pi^{w}_{s,d}(t)], \tag{S.16}$$

where α_1 and α_2 are regression coefficients estimated in [11, 13] (Table S.1). The prevalence of adult female worms $\pi^{W}_{s,d}(t)$ was calculated assuming that worms are distributed among hosts according to a negative binomial distribution with mean $W_{s,d}(t,a)$ and overdispersion parameter k_W,

$$\pi^{W}_{s,d}(t) = 1 - \left[1 + \frac{W_{s,d}(t)}{k_W}\right]^{-k_W}, \tag{S.17}$$

where $k_W = 0.35$ as estimated in [14]. The prevalence of troublesome itch was related to female adult worms because the association between the presence of microfilariae and troublesome itch does not hold during ivermectin treatment; the reduction in prevalence of itch is smaller and more delayed than the drop in microfilarial prevalence and load [11, 13, 15]. Due to fact that the EpiOncho model-derived prevalence of female worms for a given endemicity level resulted in a higher than expected prevalence of onchocercal itch (Figure S2a), it was necessary to add an adjustment factor ($\alpha_3[\pi^{w}_{s,d}(t)]$ with expression $1 + 1.8\exp\left\{-\left[\pi^{w}_{s,d}(t) - 71.5\right]0.3\right\}$ as in Table S1) to equation S.16 (Figure S2b).

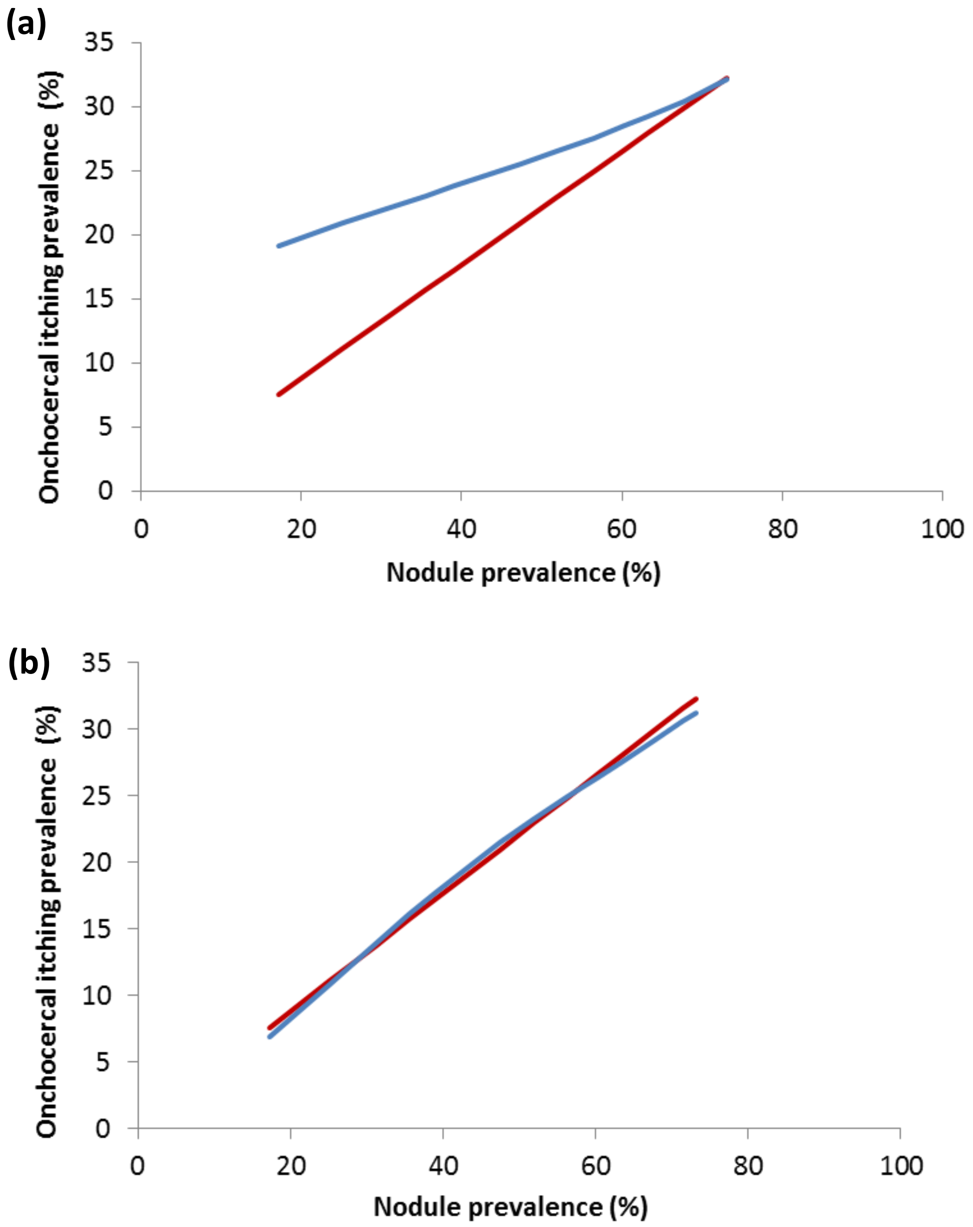

Figure S2. Comparison of onchocercal itching prevalence vs. nodule prevalence without (a) and with (b) the adjustment α_3. The red line denotes the expected onchocercal itching prevalence (based on a regression between nodule and itching prevalence presented in [11]), and the blue line denotes EpiOncho model-projected prevalence of onchocercal itch. The adjustment α_3 in eqn. (S.16) was parameterised by minimising the sum of squares between the expected (red line) and the model-projected (blue line) onchocercal itching prevalence.

The number of baseline troublesome itch cases per stratum, $T'_{s,d}(t)$, is the product of the baseline prevalence of troublesome itch, the proportion of individuals of each sex, q_s, compliance group, η_d, and the population size *P*,

$$T'_{s,d}(t) = \pi'^{T}_{s,d}(t) q_s \eta_d P. \tag{S.18}$$

The total number of baseline troublesome itch cases is then given by summing over the sex and compliance strata,

$$T'(t) = \sum_s \sum_d T'_{s,d}(t). \tag{S.19}$$

S.1.5.1 *Therapeutic Effect of Ivermectin on Troublesome Itch*

The number of baseline cases of troublesome itch is multiplied by a factor $1-\tau_d(t)$ to yield the total number of troublesome itch cases, $T(t)$,

$$T(t) = T'(t)\left[1-\tau_d(t)\right], \tag{S.20}$$

and the prevalence of troublesome itch is given by,

$$\pi^T(t) = \frac{T(t)}{P}. \tag{S.21}$$

Parameter $\tau_d(t)$ captures the observed therapeutic effect of ivermectin in reducing the average year-round baseline prevalence of troublesome itch by 47% in individuals treated annually and by 52% in those treated biannually, an effect which was assumed to develop gradually during the first six months of the first treatment round [15] and which was consistent with the results of a multicentre study conducted by the African Programme for Onchocerciasis Control (APOC) [16]. These reductions are not assumed to act cumulatively with each

treatment round but rather over the timespan of ivermectin distribution. It was further assumed that individuals treated every other year experience a 10% reduction in troublesome itch in the year they are not treated, due to the residual effects of ivermectin on cutaneous pathologies. This was estimated by the difference between the reduction of itch prevalence at 12 and 15 months after a single treatment and assumes the rate of recovery is constant from 15 months onwards [15]. Long-term reductions in the prevalence of troublesome itch are not just caused by the therapeutic activity of ivermectin but rather by its community-level suppression of transmission which leads to a gradual decrease in the prevalence of adult female worms.

S.1.6 *Excess Mortality*

The excess mortality due to onchocerciasis is assumed to occur via two processes; an additional risk of mortality among individuals suffering from onchocerciasis-related vision loss [8, 17], and an additional risk of mortality (independent of the former) among infected individuals with high microfilarial loads [18, 19]. To calculate the incidence of (excess) mortality associated with onchocerciasis these additional risks were multiplied by the age-specific background mortality (hazard) rate, $\xi(a)$,

$$\xi(a) = -\frac{d}{da}\ln\left[\varepsilon(a)\right], \qquad \text{(S.22)}$$

where $\varepsilon(a)$ is the survivorship function,

$$\varepsilon(a) = \begin{cases} \exp(-\omega_1 a) & \text{for } a < 5 \text{years}, \\ \omega_2 + \omega_3 a^2 + \omega_4 a & \text{for } a \geq 5 \text{years}. \end{cases} \qquad \text{(S.23)}$$

The regression coefficients $\omega_1, \omega_2, \omega_3$ and ω_4 were estimated by fitting $\varepsilon(a)$ to the same data that were used to parameterise the host survivorship function in ONCHOSIM [20] (Figure S.3). This makes our estimates of the excess mortality due to onchocerciasis comparable with other modelling studies.

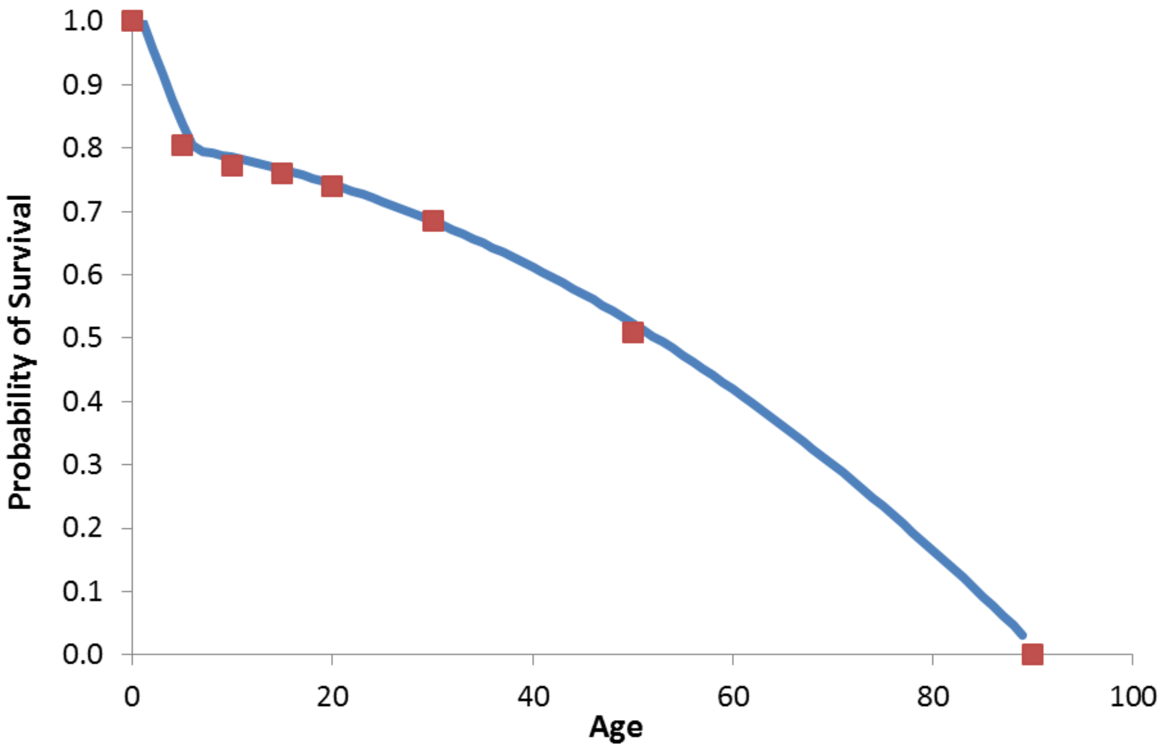

Figure S3. Human host survivorship function. The blue line represents the fitted model (Equation (S.23)) and the red squares the host survivorship data (proportion of hosts surviving to a particular age) presented in [20]. The model was fitted using non-linear least squares regression yielding parameter estimates ω_1 = 0.04, ω_2 = 0.81, ω_3 = -7.7×10^{-5} and ω_4 = 2.1×10^{-3} (Table S.1).

S.1.6.1 *Excess Mortality Associated with Vision Loss*

It was assumed that the relative risk of mortality among blind individuals, E^B, and among those with visual impairment, E^V, were, respectively, 2.5 and 1.5 according to [8]. The incidence of excess mortality associated with blindness, $D^B_{s,d}(t,a)$ and visual impairment, $D^V_{s,d}(t,a)$ are given by,

$$D^B_{s,d}(t,a) = B_{s,d}(t,a)\xi(a)\left(E^B - 1\right), \quad \text{(S.24)}$$

and

$$D^V_{s,d}(t,a) = V_{s,d}(t,a)\xi(a)\left(E^V - 1\right). \quad \text{(S.25)}$$

By analogy with the derivation of the incidence of blindness due to onchocerciasis given by Equations (S.4) to (S.7), the minus 1 term on the RHS of Equation (S.25) and Equation (S.26) adjusts the relative risks to give the incidence of mortality *due to* onchocerciasis, as opposed to the *total* incidence of mortality.

S.1.6.2 *Excess Mortality Associated with Microfilarial Load*

A density-dependent association has been quantified between excess human mortality and microfilarial load [18, 19]. This was incorporated into the model using a published non-linear, host age-dependent association between the relative risk of mortality, $E^M_{s,d}(t,a)$, in those infected (but without vision loss) and their past (lagged by two years) microfilarial load per skin snip denoted as $M'_{s,d}$, as opposed to $M_{s,d}$ for mf/mg, see also Equation (S.8),

$$E^{M}_{s,d}(t,a) = \exp\left\{ f\left[M'_{s,d}(t-2,a)\right] a^{\beta_4} \right\} \pi^{M}_{s,d}(t-2,a)\,. \tag{S.26}$$

Here $f\left[M'_{s,d}(t-2,a)\right]$ describes a sigmoid functional form,

$$f\left[M'_{s,d}(t-2,a)\right] = \frac{\beta_1 M'_{s,d}(t-2,a)^{\beta_3}}{1+\beta_2 M'_{s,d}(t-2,a)^{\beta_3}}\,, \tag{S.27}$$

where $\beta_1, \beta_2, \beta_3$ and β_4 are regression parameters as estimated in [19] (Table S.1). The prevalence term in Equation (S.26), $\pi^{M}_{s,d}(t-2,a)$, ensures that the relative risk of excess mortality associated with high microfilarial load only applies to those infected and not to the whole population.

The incidence of excess mortality associated with the mean number of microfilariae per skin snip, $D^{M}_{s,d}(t,a)$, is given by

$$D^{M}_{s,d}(t,a) = \left\{P_{s,d}(a) - \left[B_{s,d}(t,a) + V_{s,d}(t,a)\right]\right\} \xi(a) \left[E^{M}_{s,d}(t,a) - 1\right]. \tag{S.28}$$

The term $P_{s,d}(a) - \left[B_{s,d}(t,a) + V_{s,d}(t,a)\right]$ on the RHS of Equation (S.28) ensures that the incidence of excess mortality does not apply to prevalent cases of blindness or visual impairment; such individuals suffer a separate excess risk of mortality as described by Equation (S.24) and Equation (S.25).

S.1.6.3 *Total Incidence of Excess Mortality*

The total incidence of excess mortality aassociated with onchocerciasis—either via blindness (Equation (S.24)), visual impairment (Equation (S.25)) or via the direct association with microfilarial load (Equation (S.28))—is the sum of the component incidence rates,

$$D_{s,d}(t,a) = D^{B}_{s,d}(t,a) + D^{V}_{s,d}(t,a) + D^{M}_{s,d}(t,a) \quad . \tag{S.29}$$

S2. Disability Adjusted Life Years

Disability-adjusted life years (DALYs) are a time-based measure of disease burden accounting for healthy years of life lived with disability (YLDs) and years of life lost (YLLs) due to premature mortality [21].

The YLDs were calculated by multiplying the number of cases of blindness, visual impairment and troublesome itch by their respective disability weights h^{B}, h^{V}, h^{T} (provided by the Global Burden of Disease (2004) study [22]),

$$\text{YLDs}(t) = B(t)h^{B} + V(t)h^{V} + T(t)h^{T} \quad . \tag{S.30}$$

The equivalent disability weights from the Global Burden of Disease (2010) study [23] were not used, because (other than blindness) they are stratified by severity levels. For example, the skin disease disability weights were stratified into three “disfigurement levels”, and visual impairment into “mild”, “moderate”, and “severe”. Without more detailed definitions, it was not possible to relate such levels to the modelled disease sequelae.

The YLLs were calculated using the ‘period expected years of life lost’ method [24], where the duration of life lost is the local future life-expectancy of individuals at each age, $\Xi(a)$, minus there current age, multiplied by the incidence of mortality, $D_{s,d}(t,a)$,

$$\text{YLLs}(t) = \sum_{s}\sum_{d}\int_{a}\left[\Xi(a) - a\right]D_{s,d}(t,a)\,da \, \cdot \tag{S.31}$$

The age-specific future life-expectancy is derived directly from the host survivorship function, $\varepsilon(a)$,

$$\Xi(a) = \frac{\int_{u=a}^{u=\infty} \varepsilon(u)\, du}{\varepsilon(a)} . \quad \text{(S.32)}$$

The YLLs were discounted at a rate of 3% per year in line with WHO guidelines [24].

The total DALY burden is the sum of YLDs and the YLLs,

$$\text{DALYs}(t) = \text{YLDs}(t) + \text{YLLs}(t) . \quad \text{(S.33)}$$

In line with the methodology outlined in the Disease Control Priorities Project: Priorities in Health report [25], we did not apply any age weighting (whereby healthy life lived at younger and older ages is given a lower weight than that at productive adult ages) to the DALYs es timates [25]. Definitions and values of parameters are given in Supporting Table S2.

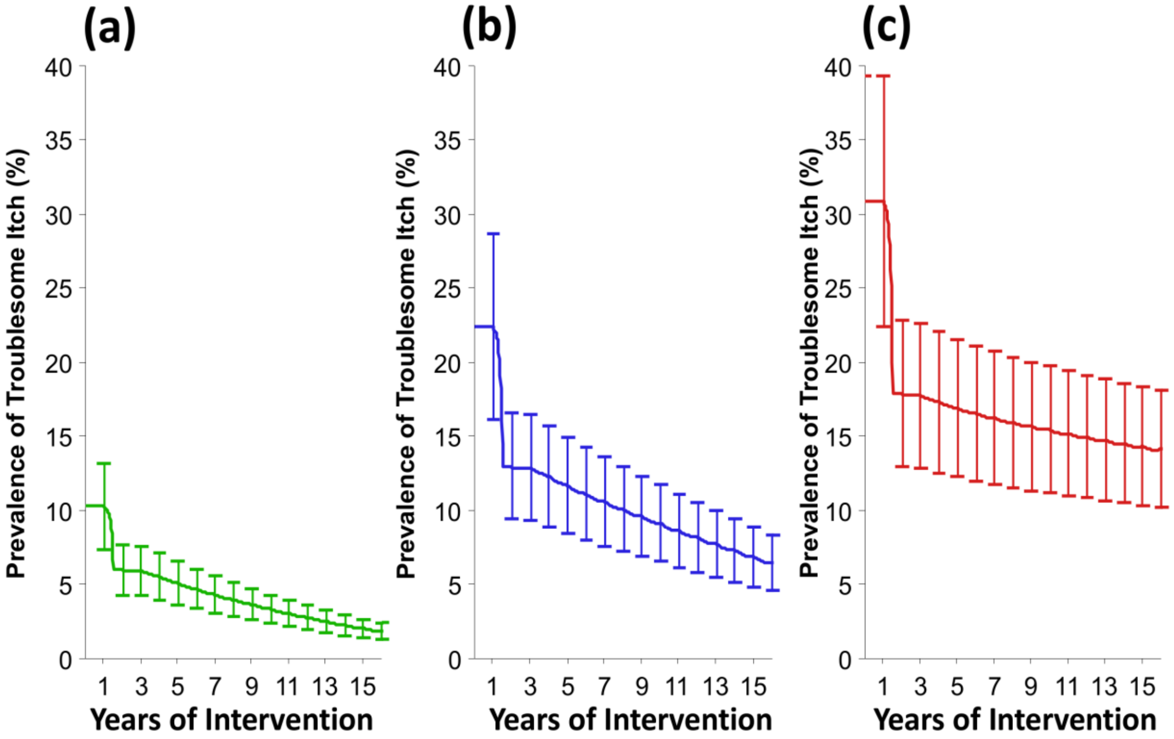

Figure S4. Sensitivity of the impact of annual ivermectin distribution on troublesome itch. Error bars denote varying the association between prevalence of adult female worms and troublesome itch described in eqn. (S.16) by varying regression coefficient α_2 by plus or minus 25%. Panels (a), (b) and (c) correspond to, respectively, a baseline endemicity of 40%, 60% and 80% microfilarial prevalence. Results shown assume a therapeutic coverage of 80%, 0.1% of systematic non-compliance, perennial transmission, and a 7% cumulative reduction in microfilarial production by female adult worms per ivermectin dose.

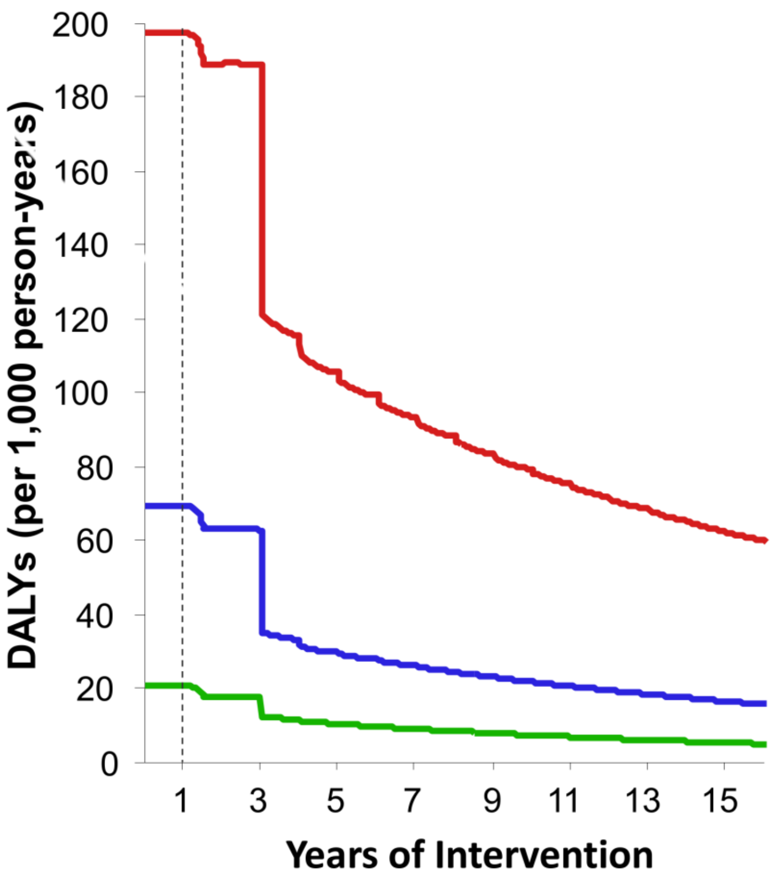

Figure S5. Impact of annual ivermectin distribution on the DALY burden associated with onchocerciasis in savannah areas of Africa. Red, blue and green lines correspond to, respectively, a baseline endemicity of 80%, 60% and 40% microfilarial prevalence. The commencement of the intervention at year 1 is represented by the vertical dashed line. Results shown assume a therapeutic coverage of 80%, 0.1% of systematic non-compliance, perennial transmission, and a 7% cumulative reduction in microfilarial production by female adult worms per ivermectin dose.

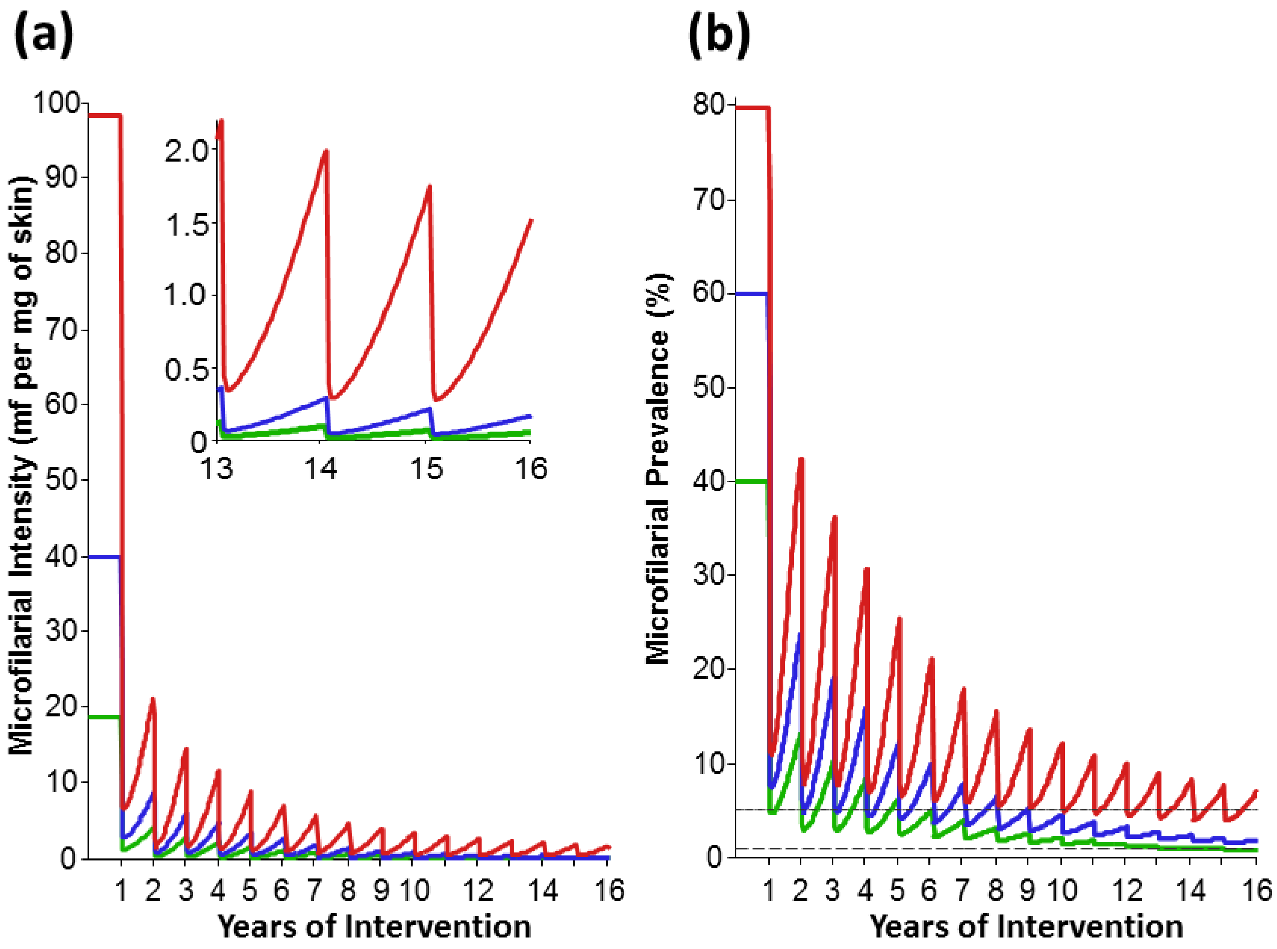

Figure S6. Impact of annual ivermectin distribution on: (a) microfilarial intensity and (b) microfilarial prevalence when assuming a stronger anti-macrofilarial action. The red, blue and green lines correspond, respectively, to a baseline endemicity of 80%, 60% and 40% microfilarial prevalence. Microfilarial intensity is quantified as the mean microfilarial load per mg of skin in those aged ≥ 20 years. The dashed horizontal lines illustrate the upper and lower bounds (5% and 1% prevalence) of the current operational thresholds for cessation of treatment, namely an observed a microfilarial prevalence below 5% in all surveyed villages and 1% in 90% of the surveyed village) [26]. Results shown assume a therapeutic coverage of 80%, 0.1% of systematic non-compliance, perennial transmission, and a 30% cumulative reduction in microfilarial production by female adult worms per ivermectin dose. The inset in Figure S4(a) zooms in microfilarial infection intensity (in the ≥20 yr of age) for the last four years of the simulated intervention programme.

References

1. Boussinesq M: **Etude épidémiologique de l'onchocercose en zone de savane camerounaise. Effets d'un traitement de masse par l'ivermectine**. PhD thesis. University of Montpellier II; 1991.
2. Basáñez MG, Boussinesq M: **Population biology of human onchocerciasis**. *Philos Trans R Soc Lond B Biol Sci* 1999, **354**(1384):809-826.
3. Prost A, Prod'hon J: **Le diagnostique de l'onchocercose. Revue critique des méthodes en usage.** *Méd Trop Mars* 1978, **38**(5):519-532.
4. Collins RC, Gonzales-Peralta C, Castro J, Zea-Flores G, Cupp MS, Richards FO, Cupp EW: **Ivermectin: reduction in prevalence and infection intensity of *Onchocerca volvulus* following biannual treatments in five Guatemalan communities.** *Am J Trop Med Hyg* 1992, **47**(2):156–169.
5. Emukah EC, Osuoha E, Miri ES, Onyenama J, Amazigo U, Obijuru C, Osuji N, Ekeanyanwu J, Amadiegwu S, Korve K *et al*: **A longitudinal study of impact of repeated mass ivermectin treatment on clinical manifestations of onchocerciasis in Imo State, Nigeria**. *Am J Trop Med Hyg* 2004, **70**(5):556-561.
6. Filipe JAN, Boussinesq M, Renz A, Collins RC, Vivas-Martinez S, Grillet ME, Little MP, Basáñez MG: **Human infection patterns and heterogeneous exposure in river blindness**. *Proc Natl Acad Sci U S A* 2005, **102**(42):15265-15270.
7. Little MP, Basáñez MG, Breitling LP, Boatin BA, Alley ES: **Incidence of blindness during the Onchocerciasis control programme in western Africa, 1971-2002**. *J Infect Dis* 2004, **189**(10):1932-1941.
8. Shibuya K, Bernard C, Ezzati M, Mathers CD: **Global burden of onchocerciasis in the year 2000: Summary of methods and data sources** World Health Organization 2006 [http://www.who.int/healthinfo/statistics/bod_onchocerciasis.pdf]
9. Remme JHF: **Global burden of onchocerciasis in 1990**. World Health Organization 2004 [http://www.who.int/healthinfo/global_burden_disease/Onchocerciasis%201990.pdf]
10. World Health Organization: **Visual field testing as a routine during ophthalmological examination of EPI villages**. Geneva: World Health Organization (OCP/EAC4.2/83.2); 1983.
11. Coffeng LE, Stolk WA, Zouré HGM, Veerman JL, Agblewonu KB, Murdoch ME, Noma M, Fobi G, Richardus JH, Bundy DAP *et al*: **African Programme for Onchocerciasis Control 1995–2015: Model-estimated health impact and cost**. *PLoS Negl Trop Dis* 2013, **7**(1):e2032.
12. Ejere H, Schwartz E, Wormald R, Evans JR: **Ivermectin for onchocercal eye disease (river blindness)**. *Cochrane Database Syst Rev* 2012 8:CD002219.
13. Habbema D, Stolk W, Veerman L, de Vlas S: **A rapid health impact assessment of APOC: Exeutive summary & technical report** APOC 2007 [http://www.who.int/apoc/publications/APOC%20rapid%20HIA_final%20report_def.pdf]
14. Bottomley C, Isham V, Collins RC, Basáñez MG: **Rates of microfilarial production by *Onchocerca volvulus* are not cumulatively reduced by multiple ivermectin treatments**. *Parasitology* 2008, **135**(13):1571-1581.
15. Brieger WR, Awedoba AK, Eneanya CI, Hagan M, Ogbuagu KF, Okello DO, Ososanya OO, Ovuga EB, Noma M, Kale OO *et al*: **The effects of ivermectin on**

onchocercal skin disease and severe itching: results of a multicentre trial. *Trop Med Int Health* 1998, **3**(12):951-961.

16. Ozoh GA, Murdoch ME, Bissek AC, Hagan M, Ogbuagu K, Shamad M, Braide EI, Boussinesq M, Noma MM, Murdoch IE *et al*: **The African Programme for Onchocerciasis Control: impact on onchocercal skin disease**. *Trop Med Int Health* 2011, **16**(7):875-883.
17. Kirkwood B, Smith P, Marshall T, Prost A: **Relationships between mortality, visual acuity and microfilarial load in the area of the Onchocerciasis Control Programme**. *Trans R Soc Trop Med Hyg* 1983, **77**(6):862-868
18. Little MP, Breitling LP, Basáñez MG, Alley ES, Boatin BA: **Association between microfilarial load and excess mortality in onchocerciasis: an epidemiological study**. *Lancet* 2004, **363**(9420):1514-1521.
19. Walker M, Little MP, Wagner KS, Soumbey-Alley EW, Boatin BA, Basáñez MG: **Density-dependent mortality of the human host in onchocerciasis: relationships between microfilarial load and excess mortality**. *PLoS Negl Trop Dis* 2012, **6**(3):e1578.
20. Habbema JDF, van Oortmarssen GJ, Plaisier AP: **The ONCHOSIM model and it use in decision support for river blindness control**. In: *Models for Infectious Human Diseases.* Edited by Isham V, Medley G. Cambridge: Cambridge University Press; 1996.
21. Murray CJL, Lopez AD: **Quantifying disability: data, methods and results**. *Bull World Health Organ* 1994, **72**(3):481-494.
22. World Health Organization: **Global Burden of Disease update 2004: disability weights for diseases and conditions.** World Health Organization 2004 [http://www.who.int/healthinfo/global_burden_disease/GBD2004_DisabilityWeights.pdf]
23. Salomon JA, Vos T, Hogan DR, Gagnon M, Naghavi M, Mokdad A, Begum N, Shah R, Karyana M, Kosen S *et al*: **Common values in assessing health outcomes from disease and injury: disability weights measurement study for the Global Burden of Disease Study 2010**. *Lancet* 2012, **380**(9859):2129-2143.
24. World Health Organization: **Making choices in health: WHO guide to cost-effectiveness analysis**. World Health Organization 2003 [http://www.who.int/choice/publications/p_2003_generalised_cea.pdf]
25. Disease Control Priorities Project: **Priorities in health**. World Bank 2006 [http://www.dcp2.org/pubs/PIH]
26. African Programme for Onchocerciasis Control: **Conceptual and operational framework of onchocerciasis elimination with ivermectin treatment**. WHO/APOC 2010 [http://www.who.int/apoc/oncho_elimination_report_english.pdf]
