# Supplementary material for: Modelling the impact of ivermectin on River Blindness and its burden of morbidity and mortality in African Savannah: EpiOncho projections
Source: Parasit Vectors. 2014 May 26;7:241. doi: 10.1186/1756-3305-7-241 (PMC4037555; doi:10.1186/1756-3305-7-241)
Supplement: Additional file 2: Table S1 — Definition and values of parameters and variables for the onchocerciasis disease model. Table S2. Definition and values of parameters for the disability-adjusted life years estimates. Table S3. The effect of the magnitude of the anti-macrofilarial effect of ivermectin on the microfilarial prevalence and intensity of onchocerciasis infection and its associated morbidity and mortality according to baseline endemicity. [file 1756-3305-7-241-S2.pdf]

Turner HC, Walker M, Churcher TS, Basáñez MG. Modelling the Impact of Ivermectin on River Blindness and its Burden of Morbidity and Mortality in African Savannah: EpiOncho Projections

Supplementary Table S1. Definition and values of parameters and variables for the onchocerciasis disease model (95% confidence limits are added wherever possible)

Symbol	Definition of variables and parameters	Expression, average value and units	Ref.
	Infection & demography		
$\pi^{M}_{s,d}(t,a)$	Microfilarial prevalence at time (*t*) and age (*a*); subscript *s* denotes host sex and subscript *d* denotes treatment compliance category	Equation (S.1)	
$M_{s,d}(t,a)$	Mean number of microfilariae per milligram of skin at time (*t*) and age (*a*); subscripts *s* and *d* as above	*Derived from transmission model*	[1-4]
$k_M\,[M_{s,d}(t,a)]$	Inverse measure of the degree of overdispersion in the distribution of skin microfilariae as a function of the mean microfilarial load at time (*t*) and age (*a*); subscripts *s* and *d* as above	Equation (S.2)	
k_0<br>k_1	Parameters determining the shape of the relationship between k_M and skin microfilarial load (Equation (S.2))	0.013 (0.010, 0.015)<br>0.025 (0.019, 0.033)	This work
η_d	Proportion of the host population in treatment compliance group *d*	–	
q_s	Proportion of the host population of sex *s*	0.45/0.55	[1]
$\rho(a)$	Truncated exponential probability density function of host age (*a*)	$\dfrac{\mu_H \exp(-\mu_H\, a)}{1-\exp(-\mu_H a_m)}$	[1]
μ_H	The net rate of population loss (due to death, emigration and other process) determining the age distribution of the population	0.04 yr^{-1}	[1]
a_m	Maximum recorded human age in the reference population of northern Cameroon	80 yr	[1]
P	Total population size for accuracy of numerical integration	100,000	

Supplementary Table S1. Continued

Symbol	Definition of variables and parameters	Expression, average value and units (±95% CI)	Ref.
	Blindness		
$\nu_{s,d}(t,a)$	Incidence of blindness due to onchocerciasis at time (*t*) and age (*a*); subscript *s* denotes host sex and subscript *d* denotes treatment compliance category	Equation (S.4)	
$\nu'_{s,d}(t,a)$	The background incidence of blindness at time (*t*) and age (*a*); subscripts *s* and *d* as above	Equation (S.5)	
$\nu^{T}_{s,d}(t,a)$	The total incidence of blindness at time (*t*) and age (*a*); subscripts *s* and *d* as above	Equation (S.6)	
$r_{s,d}(t,a)$	Relative risk of blindness incidence at time (*t*) and age (*a*); subscripts *s* and *d* as above	Equation (S.8)	[5]
γ_1	Microfilarial load regression coefficient for the relative risk of blindness incidence	$1.0\text{x}10^{-2}$ ($0.85\text{x}10^{-2}$, $1.16\text{x}10^{-2}$)	[5]
$\gamma_{0_s}(a)$	Background incidence of blindness i.e. incidence of blindness not associated with onchocerciasis at age (*a*), subscript *s* denotes host sex	–	[5]
$M'_{s,d}(t,a)$	Mean number of microfilariae per skin snip (with an average skin snip sample weight of 1.7mg [6]) at time (*t*) and age (*a*); subscripts *s* and *d* as above	–	
$B^{T}_{s,d}(t,a)$	The total number of blindness cases at time (*t*) and age (*a*); subscripts *s* and *d* as above	Equation (S.9)	
$B_{s,d}(t,a)$	Number of blindness cases due to onchocerciasis at time (*t*) and age (*a*); subscripts *s* and *d* as above	Equation (S.10)	
$\pi^{B}(t)$	Overall prevalence of blindness due to onchocerciasis at time (*t*)	Equation (S.12)	
	Visual impairment		
$V_{s,d}(t,a)$	Number of visual impairment cases due to onchocerciasis at time (*t*) and age (*a*); subscripts *s* and *d* as above	Equation (S.13)	
$\pi^{V}(t)$	Prevalence of visually impairment due to onchocerciasis at time (*t*)	Equation (S.14)	

Supplementary Table S1. Continued

Symbol	Definition of variables and parameters	Expression, average value and units	Ref.
	Troublesome itch		
$\pi'^{T}_{s,d}(t)$	Baseline prevalence of troublesome itch due to onchocerciasis at time (*t*); subscript *s* denotes host sex and subscript *d* denotes treatment compliance category	Equation (S.16)	[7, 8]
$\pi^{w}_{s,d}(t)$	Prevalence of female worms at time (*t*); subscripts *s* and *d* as above	Equation (S.17)	
α_1<br>α_2	Coefficients describing the shape of the relationship between troublesome itch and female adult worms	-0.043<br>-0.46	[7, 8]
$\alpha_3[\pi^{w}_{s,d}(t)]$	Adjustment factor to account for the difference in the EpiOncho-estimated prevalence of female worms and onchocercal itching for a given endemicity level (parameterization described in Figure S2).	$1+1.8\exp\left\{-\left[\pi^{w}_{s,d}(t)-71.5\right]0.3\right\}$	This work
$W_{s,d}(t)$	Mean number of female adult worms per person at time (*t*); subscripts *s* and *d* as above	*Derived from transmission model*	[1-4]
k_w	Inverse measure of degree of overdispersion in the distribution of female worms among hosts	0.35 (range of reported 95% CL:0.29, 0.46)	[9]
$T'_{s,d}(t)$	Number of baseline cases of troublesome itch due to onchocerciasis at time (*t*); subscripts *s* and *d* as above	Equation (S.18)	
$T(t)$	Number of cases of troublesome itch due to onchocerciasis at time (*t*)	Equation (S.20)	
$\tau_d(t)$	Average year-round reduction in the prevalence of itch associated with annual ivermectin at time (*t*); subscript *d* denotes treatment compliance category	See Supplementary Text, S.1.5.1. *Therapeutic Effect of Ivermectin on Troublesome Itch*	[10]
$\pi^{T}(t)$	Prevalence of troublesome itch due to onchocerciasis at time (*t*)	Equation (S.21)	

Supplementary Table S1. Continued

Symbol	Definition of variables and parameters	Expression, average value and units (±95% CI)	Ref
	Excess Mortality		
$\xi(a)$	Per capita background death rate of humans at age (a)	Equation (S.22)	
$\mathcal{E}(a)$	Host survivorship function	Equation (S.23)	[11]
ω_1<br>ω_2<br>ω_3<br>ω_4	Regression coefficients for the host survivorship function	0.04<br>0.81<br>-7.7×10^{-5}<br>-0.0021	
E^B	The relative risk of mortality associated with blindness	2.5	[12]
E^V	The relative risk of mortality associated with visually impairment	1.5	[12]
$D^B_{s,d}(t,a)$	Incidence of excess mortality due to blindness at time (t) and age (a); subscript s denotes host sex and d denotes treatment compliance category	Equation (S.24)	
$D^V_{s,d}(t,a)$	Incidence of excess mortality due to visual impairment at time (t) and age (a); subscripts s and d as above	Equation (S.25)	
$E^M_{s,d}(t,a)$	The relative risk of mortality associated with high microfilarial loads at time (t) and age (a); subscripts s and d as above	Equation (S.26)	[13]
$f[M'_{s,d}(t-2,a)]$	Function describing the relationship between relative risk of mortality and microfilarial load per skin snip; subscripts s and d as above	Equation (S.27)	[13]
β_1<br>β_2<br>β_3<br>β_4	Regression coefficients of the function describing the relative risk of mortality associated with high microfilarial loads	1.8 (1.2, 2.8)<br>1.8 (1.0, 3.1)<br>2.5 (1.8, 3.5)<br>-0.59 (-0.72, -0.45)	[13]
$D^M_{s,d}(t,a)$	Incidence of excess mortality associated with high microfilarial loads at time (t) and age (a); subscripts s and d as above	Equation (S.28)	
$D_{s,d}(t,a)$	Incidence of excess mortality due to onchocerciasis at time (t) and age (a); subscripts s and d as above	Equation (S.29)	

Supplementary Table S2- Definition and values of parameters for the disability adjusted life years estimates

Symbol	Definition of variables and parameters	Expression, average value and units	Ref.
h^B	Blindness disability weight	0.59	[14]
h^V	Visual impairment disability weight	0.17	[14]
h^T	Troublesome itching disability weight	0.068	[14]
YLDs(t)	Years of life with disability due to onchocerciasis at time (t)	Equation (S.30)	
YLLs(t)	Years of life lost due to onchocerciasis at time (t)	Equation (S.31)	
$\Xi(a)$	Age specific life expectancy at age (a)	Equation (S.32)	[11]
DALYs(t)	Disability adjusted life years burden due to onchocerciasis at time (t)	Equation (S.33)	

Table S3. The effect of the magnitude of the anti-macrofilarial effect of ivermectin on the microfilarial prevalence and intensity of onchocerciasis infection and its associated morbidity and mortality according to baseline endemicity

**Pre-control endemicity‡**	**Mesoendemic**			**Hyperendemic**			**Highly hyperendemic**		
**Cumulative per dose reduction in mf production**	**7%**	**30%**	**%† change**	**7%**	**30%**	**%† change**	**7%**	**30%**	**%† change**
Skin microfilarial prevalence§ (%)	1.84	0.78	58%	4.74	1.8	62%	16.69	6.92	59%
Microfilarial intensity§ (mf/mg)	0.49	0.06	88%	1.31	0.19	85%	5.47	1.59	71%
Blindness prevalence§ (%)	0.297	0.296	0.30%	0.913	0.91	0.30%	4.13	4.12	0.20%
Visual impairment prevalence§ (%)	0.4	0.39	2.50%	1.223	1.221	0.20%	5.54	5.52	0.40%
Troublesome itch prevalence§ (%)	1.80	1.34	26%	6.42	2.71	27%	14.09	12.61	10%
Excess mortality annual incidence§ (per 1000)	0.0818	0.0815	0.40%	0.26	0.25	4%	1.13	1.1	3%

§ Values correspond to model outputs 12 months after the 15th annual ivermectin treatment assuming perennial transmission, an overall treatment coverage of 80% (high coverage), and 0.1% of systematic non-compliance (high treatment adherence). Microfilarial infection intensity is quantified as arithmetic mean microfilarial load per mg of skin in those aged ≥ 20 years.† Proportional (percent) reduction in parasitological, morbidity and mortality indicators relative to the lower (7%) cumulative reduction in the rate of microfilarial production by adult female worms.

‡ Pre-control microfilarial prevalence as in Table 1 of the main text.

Turner HC, Walker M, Churcher TS, Basáñez MG. Modelling the Impact of Ivermectin on River Blindness and its Burden of Morbidity and Mortality in African Savannah: EpiOncho Projections

1. Filipe JAN, Boussinesq M, Renz A, Collins RC, Vivas-Martinez S, Grillet ME, Little MP, Basáñez MG: **Human infection patterns and heterogeneous exposure in river blindness**. *Proc Natl Acad Sci U S A* 2005, **102**(42):15265-15270.
2. Churcher TS, Basáñez MG: **Density dependence and the spread of anthelmintic resistance**. *Evolution* 2008, **62**(3):528-537.
3. Turner HC, Churcher TS, Walker M, Osei-Atweneboana MY, Prichard RK, Basáñez MG: **Uncertainty surrounding projections of the long-term impact of ivermectin treatment for human onchocerciasis**. *PLoS Negl Trop Dis* 2013, **7**(4):e2169.
4. Basáñez MG, Boussinesq M: **Population biology of human onchocerciasis**. *Philos Trans R Soc Lond B Biol Sci* 1999, **354**(1384):809-826.
5. Little MP, Basáñez MG, Breitling LP, Boatin BA, Alley ES: **Incidence of blindness during the Onchocerciasis control programme in western Africa, 1971-2002**. *J Infect Dis* 2004, **189**(10):1932-1941.
6. Collins RC, Gonzales-Peralta C, Castro J, Zea-Flores G, Cupp MS, Richards FO, Cupp EW: **Ivermectin: reduction in prevalence and infection intensity of *Onchocerca volvulus* following biannual treatments in five Guatemalan communities.** *Am J Trop Med Hyg* 1992, **47**(2):156–169.
7. Habbema D, Stolk W, Veerman L, de Vlas S: **A rapid health impact assessment of APOC: Exeutive summary & technical report** APOC 2007 [http://www.who.int/apoc/publications/APOC%20rapid%20HIA_final%20report_def.pdf]
8. Coffeng LE, Stolk WA, Zouré HGM, Veerman JL, Agblewonu KB, Murdoch ME, Noma M, Fobi G, Richardus JH, Bundy DAP *et al*: **African Programme for Onchocerciasis Control 1995–2015: Model-estimated health impact and cost**. *PLoS Negl Trop Dis* 2013, **7**(1):e2032.
9. Bottomley C, Isham V, Collins RC, Basáñez MG: **Rates of microfilarial production by *Onchocerca volvulus* are not cumulatively reduced by multiple ivermectin treatments**. *Parasitology* 2008, **135**(13):1571-1581.
10. Brieger WR, Awedoba AK, Eneanya CI, Hagan M, Ogbuagu KF, Okello DO, Ososanya OO, Ovuga EB, Noma M, Kale OO *et al*: **The effects of ivermectin on onchocercal skin disease and severe itching: results of a multicentre trial**. *Trop Med Int Health* 1998, **3**(12):951-961.
11. Habbema JDF, van Oortmarssen GJ, Plaisier AP: **The ONCHOSIM model and it use in decision support for river blindness control**. In: *Models for Infectious Human Diseases.* Edited by Isham V, Medley G. Cambridge: Cambridge University Press; 1996.
12. Shibuya K, Bernard C, Ezzati M, Mathers CD: **Global burden of onchocerciasis in the year 2000: Summary of methods and data sources** World Health Organization 2006 [http://www.who.int/healthinfo/statistics/bod_onchocerciasis.pdf]
13. Walker M, Little MP, Wagner KS, Soumbey-Alley EW, Boatin BA, Basáñez MG: **Density-dependent mortality of the human host in onchocerciasis: relationships between microfilarial load and excess mortality**. *PLoS Negl Trop Dis* 2012, **6**(3):e1578.
14. World Health Organization: **Global Burden of Disease update 2004: disability weights for diseases and conditions.** World Health Organization 2004 [http://www.who.int/healthinfo/global_burden_disease/GBD2004_DisabilityWeights.pdf]
